# A molecular comparison of [Fe-S] cluster-based homeostasis in *Escherichia coli* and *Pseudomonas aeruginosa*

**DOI:** 10.1128/mbio.01206-24

**Published:** 2024-10-03

**Authors:** Alessandra Lo Sciuto, Francesca D'Angelo, Maria Concetta Spinnato, Pierre Simon Garcia, Shirley Genah, Cervoni Matteo, Emmanuel Séchet, Ehud Banin, Frédéric Barras, Francesco Imperi

**Affiliations:** 1 Department of Science, Roma Tre University, Rome, Italy; 2 Department of Microbiology, Stress Adaptation and Metabolism Unit, UMR CNRS 6047, Université Paris-Cité, Institut Pasteur, Paris, France; 3 Department of Biology and Biotechnology “Charles Darwin”, Sapienza University, Rome, Italy; 4 Institute for Nanotechnology and Advanced Materials, Bar-Ilan University, Ramat Gan, Israel; 5 IRCCS Fondazione Santa Lucia, Rome, Italy; 6 NBFC, National Biodiversity Future Center, Palermo, Italy; National Institute of Child Health and Human Development (NICHD), Bethesda, Maryland, USA

**Keywords:** stress adaptation, *Escherichia coli*, *Pseudomonas aeruginosa*, iron-sulfur biogenesis

## Abstract

Iron-sulfur [Fe-S] clusters are essential protein cofactors allowing bacteria to perceive environmental redox modification and to adapt to iron limitation. *Escherichia coli*, which served as a bacterial model, contains two [Fe-S] cluster biogenesis systems, ISC and SUF, which ensure [Fe-S] cluster synthesis under balanced and stress conditions, respectively. However, our recent phylogenomic analyses revealed that most bacteria possess only one [Fe-S] cluster biogenesis system, most often SUF. The opportunist human pathogen *Pseudomonas aeruginosa* is atypical as it harbors only ISC. Here, we confirmed the essentiality of ISC in *P. aeruginosa* under both normal and stress conditions. Moreover, *P. aeruginosa* ISC restored viability, under balanced growth conditions, to an *E. coli* strain lacking both ISC and SUF. Reciprocally, the *E. coli* SUF system sustained growth and [Fe-S] cluster-dependent enzyme activities of ISC-deficient *P. aeruginosa*. Surprisingly, an ISC-deficient *P. aeruginosa* strain expressing *E. coli* SUF showed defects in resistance to H_2_O_2_ stress and paraquat, a superoxide generator. Similarly, the *P. aeruginosa* ISC system did not confer stress resistance to a SUF-deficient *E. coli* mutant. A survey of 120 Pseudomonadales genomes confirmed that all but five species have selected ISC over SUF. While highlighting the great versatility of bacterial [Fe-S] cluster biogenesis systems, this study emphasizes that their contribution to cellular homeostasis must be assessed in the context of each species and its own repertoire of stress adaptation functions. As a matter of fact, despite having only one ISC system, *P. aeruginosa* shows higher fitness in the face of ROS and iron limitation than *E. coli*.

**IMPORTANCE:**

ISC and SUF molecular systems build and transfer Fe-S cluster to cellular apo protein clients. The model *Escherichia coli* has both ISC and SUF and study of the interplay between the two systems established that the ISC system is the house-keeping one and SUF the stress-responding one. Unexpectedly, our recent phylogenomic analysis revealed that in contrast to *E. coli* (and related enterobacteria such as Salmonella), most bacteria have only one system, and, in most cases, it is SUF. *Pseudomonas aeruginosa* fits the general rule of having only one system but stands against the rule by having ISC. This study aims at engineering *P. aeruginosa* harboring *E. coli* systems and vice versa. Comparison of the recombinants allowed to assess the functional versatility of each system while appreciating their contribution to cellular homeostasis in different species context.

## INTRODUCTION

In their environment, bacteria are continuously thriving among fluctuating conditions, yielding to frequent unbalanced stressful situations. The capacity to sustain such sudden and frequent changes stems from the ability to detect them and to modify cellular pathways such as to reach a new homeostatic state. Most frequent stresses encountered by bacteria include those derived from reactive chemical species or those due to nutrient limitation. Reactive oxygen species (ROS) or reactive nitrogen species (RNS) and iron limitation are typical examples of such stresses, in particular, for pathogens multiplying in their host. In this context, iron-sulfur [Fe-S] clusters are of great relevance as these protein cofactors are often used by bacteria to detect and cope with ROS/RNS and iron limitation (1, 2). Moreover, [Fe-S] cluster-bound proteins are targets of ROS/RNS and iron limitation because the former might destabilize clusters and the latter could make them difficult to build.

[Fe-S] cluster bound proteins, which are involved in multiple conserved cellular processes, acquire their clusters from dedicated [Fe-S] cluster biogenesis systems (3, 4). Five [Fe-S] cluster biogenesis systems have been identified in prokaryotes, namely, NIF (NItrogen Fixation), ISC (Iron–Sulfur Cluster), SUF (mobilization of SUlFur), MIS (Minimal Isc System), and SMS (Suf-like Minimal System) (5). The NIF system is specific for nitrogenase maturation and has been well studied in the nitrogen-fixing bacterium *Azotobacter vinelandii* (6–8). The SUF and ISC systems are both present in *Escherichia coli* and shown to be responsible for the maturation of all cellular [Fe-S] cluster proteins although exhibiting differential target-specific efficiency (9–13). All [Fe-S] cluster biogenesis systems, but SMS, include a cysteine desulfurase (IscS, SufSE, NifS, MisS), which provides sulfur to build a [Fe-S] cluster on a scaffold protein (IscU, SufBCD, NifU, MisU, and possibly SmsBC). Then, carriers of the A-type (IscA, SufA, ErpA, NfuA) or other types of transporters (Grx, Mrp) deliver the newly synthesized [Fe-S] clusters to apo-proteins (3, 11).

Our perception of [Fe-S] cluster homeostasis is mostly derived from studies in *E. coli*. This bacterium switches from ISC under normal conditions to SUF under stress conditions thanks to two transcriptional regulators, Fur and IscR, and a non-coding RNA, RyhB. Fur and IscR act as repressors in their Fe-bound or [Fe-S] cluster-bound form, respectively. When the level of [Fe-S] clusters synthesized meets with the overall cellular demand, the [Fe-S] cluster-bound IscR represses the *isc* operon, preventing additional [Fe-S] cluster biosynthesis. The *suf* operon is under Fur repression. Under iron limitation, Fur is mainly present in the apo form and Fur repression alleviation allows activation of the *suf* operon while the non-coding RNA RyhB inhibits *iscSUA* gene translation and promotes *iscSUA* mRNA degradation. RyhB-binding site lies in the *iscR-iscSUA* intergenic region and stabilizes upstream *iscR* mRNA, allowing for a steady production of IscR under its apo-form, which binds upstream of the *suf* operon and activates its expression. Moreover, under oxidative stress, the OxyR transcriptional regulator activates the *suf* operon expression (4). Consistent with these specific regulatory patterns, the SUF system was found to be used under oxidative stress and iron limitation, while the efficiency of the ISC system appeared to be compromised under such stress conditions (4). Thus, in *E. coli*, the presence of both ISC and SUF ensures [Fe-S] cluster synthesis to be maintained under a wide array of different growth conditions in order to meet with the essentiality of [Fe-S] clusters. Yet, this model was from the beginning bound to be restricted to *E. coli* and other *Enterobacteriaceae*, such as *Salmonella* Typhimurium or *Dickeya dadantii* (14–16), as some important human pathogens were found to possess only the SUF system, such as *Mycobacterium tuberculosis* (17), *Staphylococcus aureus* (18), and *Enterococcus faecalis* (19), or the only ISC system, such as *Pseudomonas aeruginosa* (20) and *Acinetobacter baumannii* (21). Moreover, very recently, we carried out a thorough analysis of [Fe-S] cluster biogenesis system distribution in prokaryotes and found that a vast majority of bacteria have only one system, most of the time SUF (5). Hence, besides illustrating the particularity of enterobacteria [Fe-S] cluster-based biology, these observations call for a broadening of our studies in order to embrace the biodiversity emerging in the bacterial world, focusing on bacterial models equipped with different combinations of [Fe-S] cluster biogenesis systems.

The opportunistic human pathogen *P. aeruginosa* genome contains the *iscRSUA-hscBA-fdx2-iscX* operon (20, 22). The lack of a stress responding SUF system that would intervene under oxidative stress conditions as observed in *E. coli* is surprising, as *P. aeruginosa* confronts ROS produced by host phagocytes during acute and chronic infections. The *isc* operon was found to be upregulated under oxidative stress, thanks to the alleviation of the transcriptional repression mediated by IscR, which acts as a sensor of cellular [Fe-S] cluster levels under both balanced and stressful conditions (20). This suggests that ISC might be sufficient to keep up with different environmental conditions, particularly those encountered during multiplication in the host. *P. aeruginosa* was also found to synthesize the [Fe-S] cluster carriers NfuA and GrxD, which were proposed to be contributing to [Fe-S] cluster targeting to apo-clients under oxidative stress (23).

In this study, we established the essentiality of the ISC system in *P. aeruginosa* and its role in antibiotic and stress resistance; then, we tested the interchangeability between the ISC and/or SUF systems of *E. coli* and *P. aeruginosa*, performing different analyses in several growth conditions, especially by mimicking the stressful environment that *P. aeruginosa* cells handle in the host during infection. This thorough and exhaustive comparison revealed unsuspected property of each machinery, like the reduced ability of *E. coli* SUF as compared with ISC to sustain a high level of ROS-mediated stress in *P. aeruginosa* or capacity of *P. aeruginosa* ISC to rescue a strain of *E. coli* lacking both ISC and SUF. Altogether this study allows us to discuss and, in part, reassess the contribution of [Fe-S] cluster biogenesis systems to bacterial homeostasis control.

## RESULTS

### The ISC system is essential in *P. aeruginosa*

**Fig 1:**
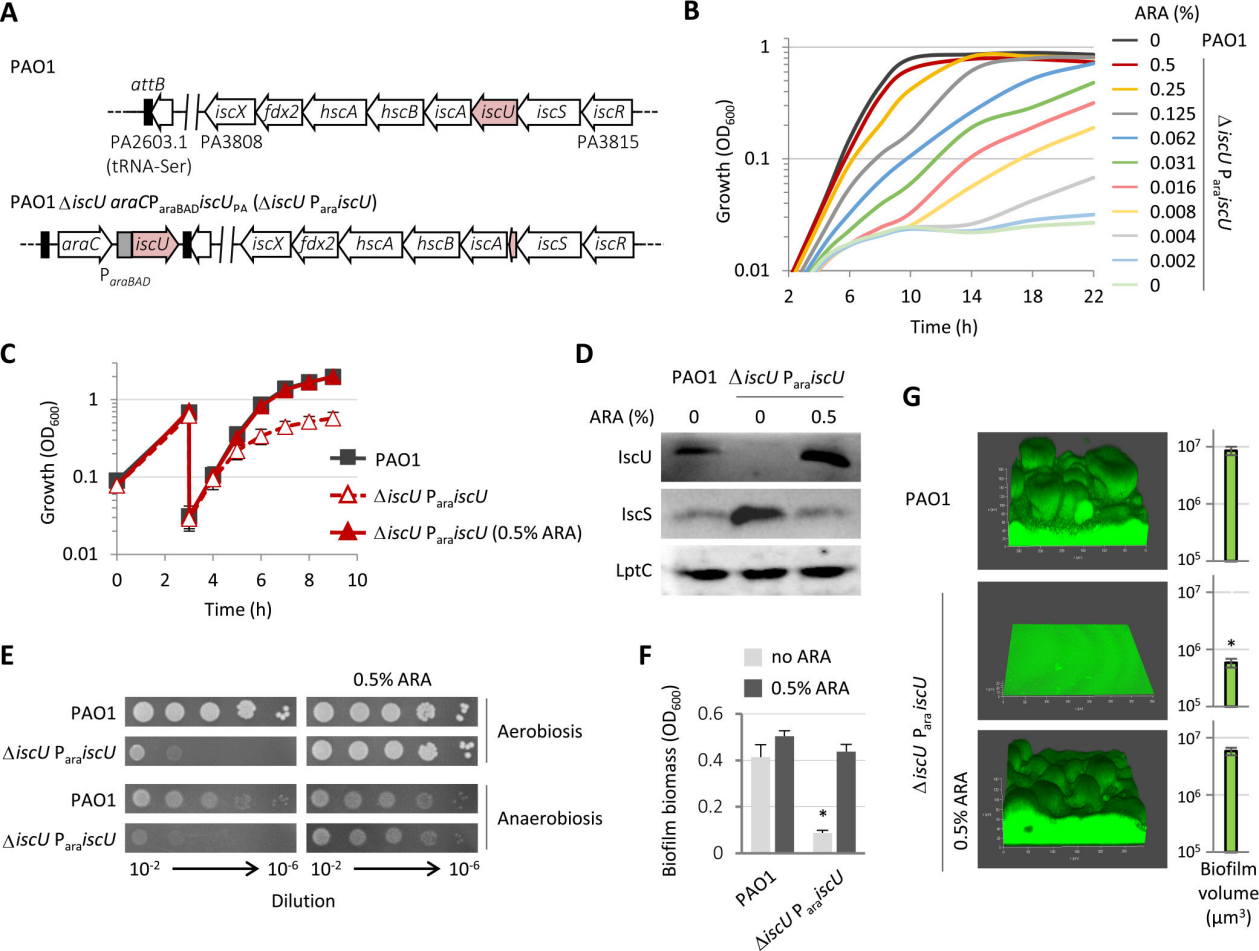
Essentiality of the ISC system in *P. aeruginosa*. (**A**) Schematic representation of the *P. aeruginosa isc* operon in the wild-type strain PAO1 and in the arabinose (ARA)-dependent Δ*iscU* P_ara_*iscU* conditional mutant. Genes are not in scale. (**B**) Growth curves of PAO1 and Δ*iscU* P_ara_*iscU* at 37°C in MH supplemented or not with increasing concentrations of ARA. (**C**) Growth of PAO1 and Δ*iscU* P_ara_*iscU* at 37°C in MH upon subsequent refreshes with or without ARA (see Materials and Methods for details). (**D**) Western blot analysis for IscU, IscS, and the loading control LptC in IscU-replete and -depleted cells obtained as shown in panel C. (**E**) Colony growth of PAO1 and Δ*iscU* P_ara_*iscU* at 37°C on MH agar plates under aerobic and anaerobic conditions. (**F and G**) Biofilm formation by PAO1 and Δ*iscU* P_ara_*iscU* in (**F**) microtiter plates or (**G**) flow cells. The asterisk indicates a statistically significant difference (*P* < 0.05) with respect to the wild type. Data are the mean (±SD) or are representative of at least three independent assays.

As [Fe-S] clusters are essential, inactivating the only system that *P. aeruginosa* possesses was predicted to be lethal. To test this, we generated a *P. aeruginosa* PAO1 conditional mutant in the *iscU* gene, encoding the ISC [Fe-S] cluster scaffold protein. This mutant, hereafter named Δ*iscU* P_ara_*iscU*, carries an arabinose-inducible copy of the *iscU* coding sequence inserted into a neutral site (*attB*) of the genome and an in-frame deletion of the endogenous *iscU* coding sequence (Fig. 1A).

Planktonic growth assays demonstrated that the Δ*iscU* P_ara_*iscU* conditional mutant cannot grow in the absence of arabinose and that the inducer promotes the growth of the mutant in a dose-dependent manner (Fig. 1B). Next, Δ*iscU* P_ara_*iscU* mutant cells were collected under non-permissive conditions, using the culturing strategy shown in Fig. 1C, and Western blot analysis confirmed the full depletion of IscU (Fig. 1D). Importantly, the IscS protein was detected, showing that deletion of the endogenous copy of *iscU* did not hamper the expression of the upstream adjacent *iscS* gene. Moreover, the intracellular levels of IscS were markedly increased in IscU-depleted cells (Fig. 1D). This was expected as the *isc* operon is under the control of the transcriptional repressor IscR, which is an IscU target and, therefore, is predicted to arise mostly in its non-repressing apo-form in IscU-depleted cells (20).

Colony growth assays showed that IscU is important for *P. aeruginosa* growth also under anaerobic conditions in the presence of nitrate as electron acceptor (Fig. 1E), presumably because the ISC system matures [Fe-S] cluster-containing nitrate reductase (24). Microtiter plate assays showed that the Δ*iscU* P_ara_*iscU* conditional mutant was also strongly impaired in biofilm formation under non-inducing conditions (Fig. 1F). Consistently, flow-cell assay confirmed that IscU-depleted cells were only able to adhere to the surface without developing mature, mushrooms-like biofilms (Fig. 1G). The biofilm biomass and structure were comparable to the wild type when the Δ*iscU* P_ara_*iscU* conditional mutant was cultured in the presence of arabinose (Fig. 1F and G), confirming that the impairment in biofilm formation was specifically due to IscU depletion. Altogether these results demonstrated that the ISC system is essential for *P. aeruginosa*.

### Effect of IscU depletion on antibiotic resistance in *P. aeruginosa*

**Fig 2:**
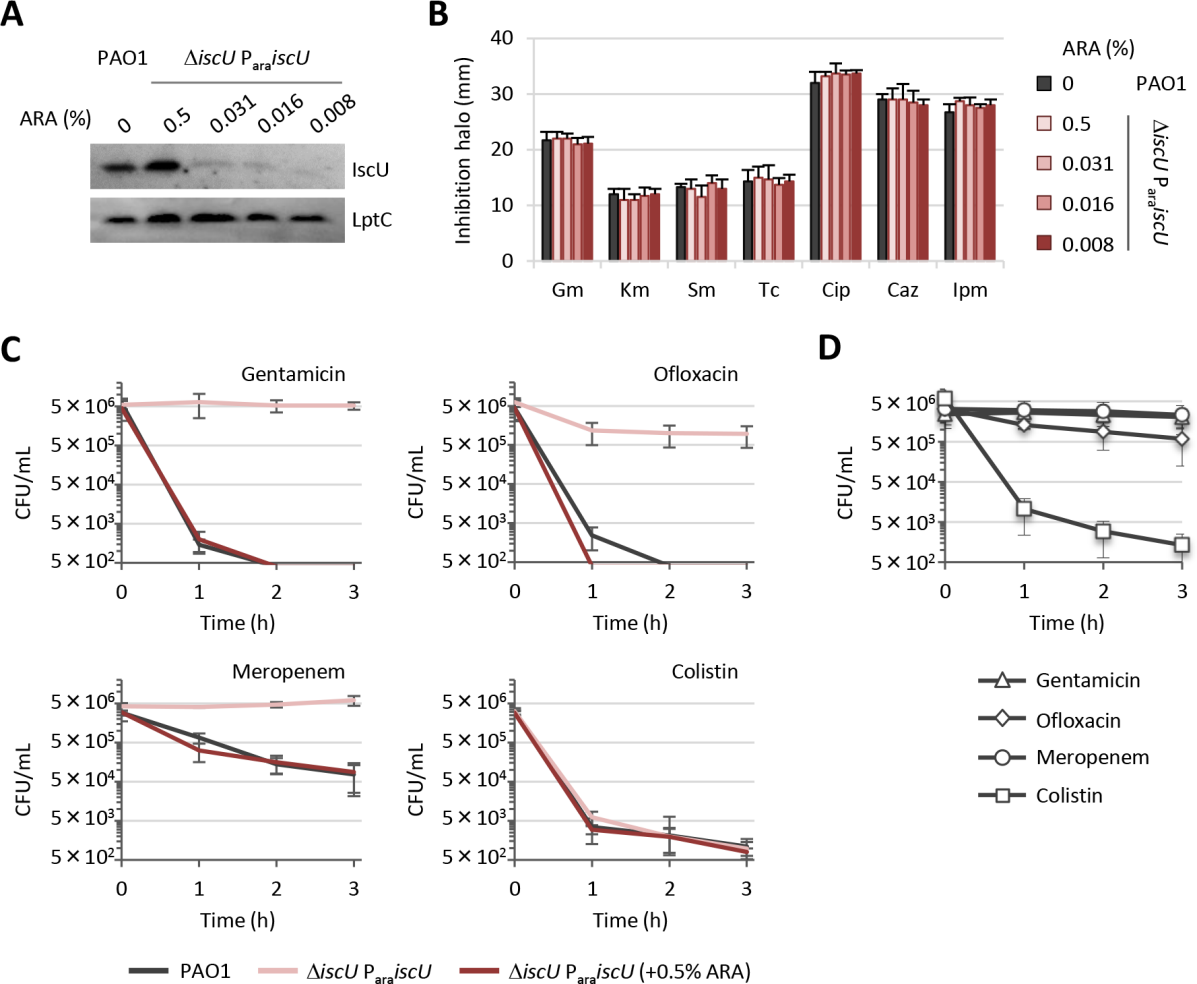
Effect of IscU depletion on *P. aeruginosa* antibiotic susceptibility. (**A**) Western blot analysis for IscU and the loading control LptC in PAO1 and Δ*iscU* P_ara_*iscU* conditional mutant cells cultured with different arabinose (ARA) concentrations. (**B**) Inhibition halos in the Kirby-Bauer disc diffusion assay of gentamicin (Gm), kanamycin (Km), streptomycin (Sm), tetracycline (Tc), ciprofloxacin (Cip), ceftazidime (Caz), and imipenem (Ipm) for PAO1 and Δ*iscU* P_ara_*iscU* cells cultured in the presence of the indicated ARA concentrations. (**C**) Survival curves of IscU-replete and -depleted cells, obtained as described in Fig. 1C, exposed to 4× MIC of gentamicin, rifampicin, meropenem or colistin, corresponding to 2 µg/mL for all antibiotics against PAO1. Curves obtained for antibiotic treatments at 1× and 2× MIC are shown in Fig. S1. (**D**) Survival curves of metabolically inactive PAO1 cells exposed to the indicated antibiotics at 4 × MIC. Cells were resuspended in saline and incubated at 4°C for 14 h (29) and then subjected to antibiotic treatment in saline. Data are the mean (±SD) or are representative of at least three independent assays.

*P. aeruginosa* causes severe infections in humans and understanding its mechanisms of antibiotic resistance is a necessity. Therefore, we investigated whether IscU depletion and the resulting impairment in [Fe-S] cluster biogenesis affects *P. aeruginosa* sensitivity to aminoglycosides. This was tested because *E. coli* cells with inefficient [Fe-S] cluster biogenesis are more resistant to aminoglycosides owing to impaired maturation of respiratory complexes and decreased level of the proton motive force (pmf) required for aminoglycoside uptake (12). First, we confirmed by Western blot analysis that IscU level in the conditional mutant decreases with decreasing concentrations of arabinose (Fig. 2A). Afterward, we performed disc diffusion assays on agar plates supplemented with arabinose at the lowest concentrations that sustain growth of the Δ*iscU* P_ara_*iscU* conditional mutant (Fig. 2B). Surprisingly, varying IscU levels did not influence resistance to aminoglycosides (gentamicin, kanamycin, streptomycin). Levels of resistance to antibiotics belonging to other classes were also unchanged (Fig. 2B). However, time-killing experiments revealed that IscU-depleted cells are much more tolerant to the aminoglycoside gentamicin, the fluoroquinolone ofloxacin, and the β-lactam meropenem than IscU-replete ones (Fig. 2C; Fig. S1). The observed pleiotropic tolerance of IscU-depleted cells to different antibiotics targeting different pathways could be due to poor metabolic activity (25, 26). This was tested using colistin, an antibiotic reported to kill both growing and resting cells (27, 28). Colistin was found to kill equally efficiently IscU-depleted and -replete cells (Fig. 2C; Fig. S1). To corroborate the link between cell metabolic state and antibiotic susceptibility, we incubated wild-type cells in saline overnight at 4°C to reduce cell metabolism (29) and then treated them with the same antibiotics. As expected, metabolically inactive *P. aeruginosa* cells acquired tolerance to all antibiotics except colistin (Fig. 2D) similar to IscU-depleted cells. These results showed that low levels of IscU and, thus, impaired production of [Fe-S] clusters yield to a pleiotropic tolerance to multiple antibiotics of different classes, likely by lowering cell metabolic activity.

### The *E. coli* SUF system can functionally replace a defective *P. aeruginosa* ISC system under normal conditions but not under stress conditions

**Fig 3:**
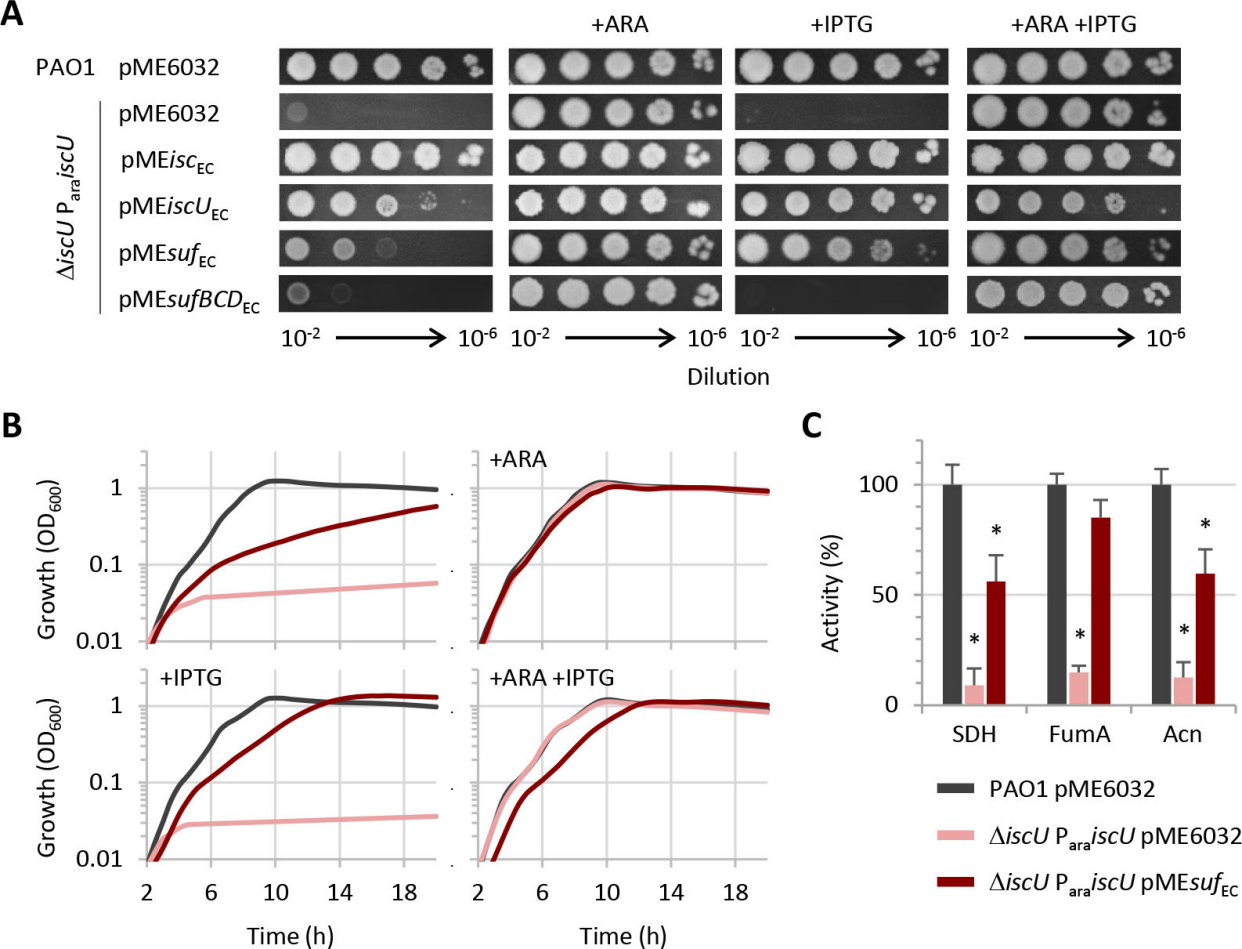
*E. coli* ISC and SUF systems functionally replace *P. aeruginosa* ISC. (**A**) Colony growth of the *P. aeruginosa* Δ*iscU* P_ara_*iscU* conditional mutant carrying the plasmid pME6032 with the entire *isc* operon (*isc*_EC_), the entire *suf* operon (*suf*_EC_), the *iscU* gene or *sufBCD* genes from *E. coli* under an IPTG-inducible promoter on MH agar plates. PAO1 and Δ*iscU* P_ara_*iscU* with the empty plasmid pME6032 were used as positive and negative controls, respectively. (**B**) Planktonic growth of PAO1 and Δ*iscU* P_ara_*iscU* carrying pME*suf*_EC_ or the empty plasmid at 37°C in MH. When indicated, arabinose (ARA) and IPTG were added at 0.5% and 0.5 mM, respectively. (**C**) Enzymatic activity, expressed as percentage relative to PAO1 pME6032, of succinate dehydrogenase (SDH), fumarase A (FumA), and aconitase (Acn) in Δ*iscU* P_ara_*iscU* pME*suf*_EC_ cultured in MH with 0.5 mM IPTG. IscU-depleted cells carrying the empty plasmid were obtained as described in Fig. 1C and used as the negative control. Data are the mean (±SD) or are representative of at least three independent assays. The asterisks indicate statistically significant differences (*P* < 0.05) with respect to the wild type carrying the empty plasmid.

*P. aeruginosa* has only ISC. Therefore, we were curious to investigate how it would behave if its ISC system was changed for a heterologous SUF system. To this aim, we cloned the entire *suf* operon (*sufABCDSE*) of *E. coli* (hereafter renamed *suf*_EC_) downstream of an IPTG-dependent promoter into the shuttle vector pME6032, yielding to the pME*suf*_EC_ plasmid, which was transformed into the *P. aeruginosa* Δ*iscU* P_ara_*iscU* conditional mutant. In parallel, we verified that the *E. coli* ISC system was able to substitute for the *P. aeruginosa* ISC. We cloned the *iscU* gene (hereafter renamed *iscU_EC_*) or the entire *isc* operon (*iscSUAhscBAfdx*, hereafter renamed *isc_EC_*) of *E. coli* downstream of the IPTG-dependent promoter into pME6032, yielding to the pME*iscU*_EC_ and pME*isc*_EC_ plasmids, which were subsequently transformed into the *P. aeruginosa* Δ*iscU* P_ara_*iscU* conditional mutant (Table S1). Colony growth assays revealed that the expression of both *E. coli* ISC and SUF restored the growth of the conditional mutant although the growth was slightly impaired in the Δ*iscU* P_ara_*iscU* conditional mutant expressing *E. coli* SUF (Fig. 3A). As expected, the expression of IscU_EC_ alone was also able to rescue the growth of the *P. aeruginosa* Δ*iscU* P_ara_*iscU* conditional mutant in the absence of arabinose (Fig. 3A). In contrast, the expression of the [Fe-S] cluster scaffold proteins of the *E. coli* SUF system (SufBCD) from the plasmid pME*sufBCD*_EC_ did not promote the growth of the Δ*iscU* P_ara_*iscU* conditional mutant (Fig. 3A), confirming that ISC and SUF components are not functionally exchangeable (3, 11). Some growth-promoting effect on IscU-depleted cells was also observed for pME*iscU*_EC_, pME*isc*_EC_, and pME*suf*_EC_ under non-inducing conditions (i.e. without IPTG) (Fig. 3A), likely because of leaky expression from pME6032 (30). This allowed us to further compare the functionality of the different [Fe-S] cluster biogenesis systems in *P. aeruginosa*. While the basal level of expression of the *isc*_EC_ operon was sufficient to fully restore *P. aeruginosa* Δ*iscU* P_ara_*iscU* growth, *E. coli* SUF failed to restore *P. aeruginosa* Δ*iscU* P_ara_*iscU* growth at low-expression levels (Fig. 3A), implying that the heterologous SUF system only partially compensates for the non-functionality of the endogenous *P. aeruginosa* ISC system. This was corroborated by (i) planktonic growth assays, which revealed a delayed growth of IscU-depleted *P. aeruginosa* cells expressing *E. coli* SUF as compared to IscU-replete controls (Fig. 3B) and (ii) biochemical assays, demonstrating that the activity of the [Fe-S] enzymes succinate dehydrogenase, aconitase, and fumarase A was slightly lower in the Δ*iscU* P_ara_*iscU* conditional mutant that expresses SUF with respect to the wild type control (Fig. 3C). Interestingly, *E. coli* SUF expression fully restored susceptibility to antibiotic killing in the Δ*iscU* P_ara_*iscU* conditional mutant (Fig. S2).

To rule out that growth restoration by *E. coli* SUF could be influenced by residual IscU expression in the conditional mutant, we generated *P. aeruginosa* PAO1 Δ*iscU* strains carrying either pME*suf*_EC_ or pME*isc*_EC_ (Table S1). Colony and planktonic growth assays demonstrated that the effect of *E. coli* ISC or SUF expression is very similar between the Δ*iscU* strain and the Δ*iscU* P_ara_*iscU* conditional mutant (Fig. S3), confirming that *E. coli* SUF is sufficient to sustain growth of ISC-deficient *P. aeruginosa* cells. The Δ*iscU* strain carrying the pME*isc*_EC_ was also used to monitor the basal level of expression from the IPTG-inducible promoter by Western blot using the anti-IscU antibody (Fig. S4). This analysis showed that the growth restoration effect of ISC expressing plasmids observed in the absence of IPTG (Fig. 3) occurs at very low levels of expression of *E. coli isc* genes.

**Fig 4:**
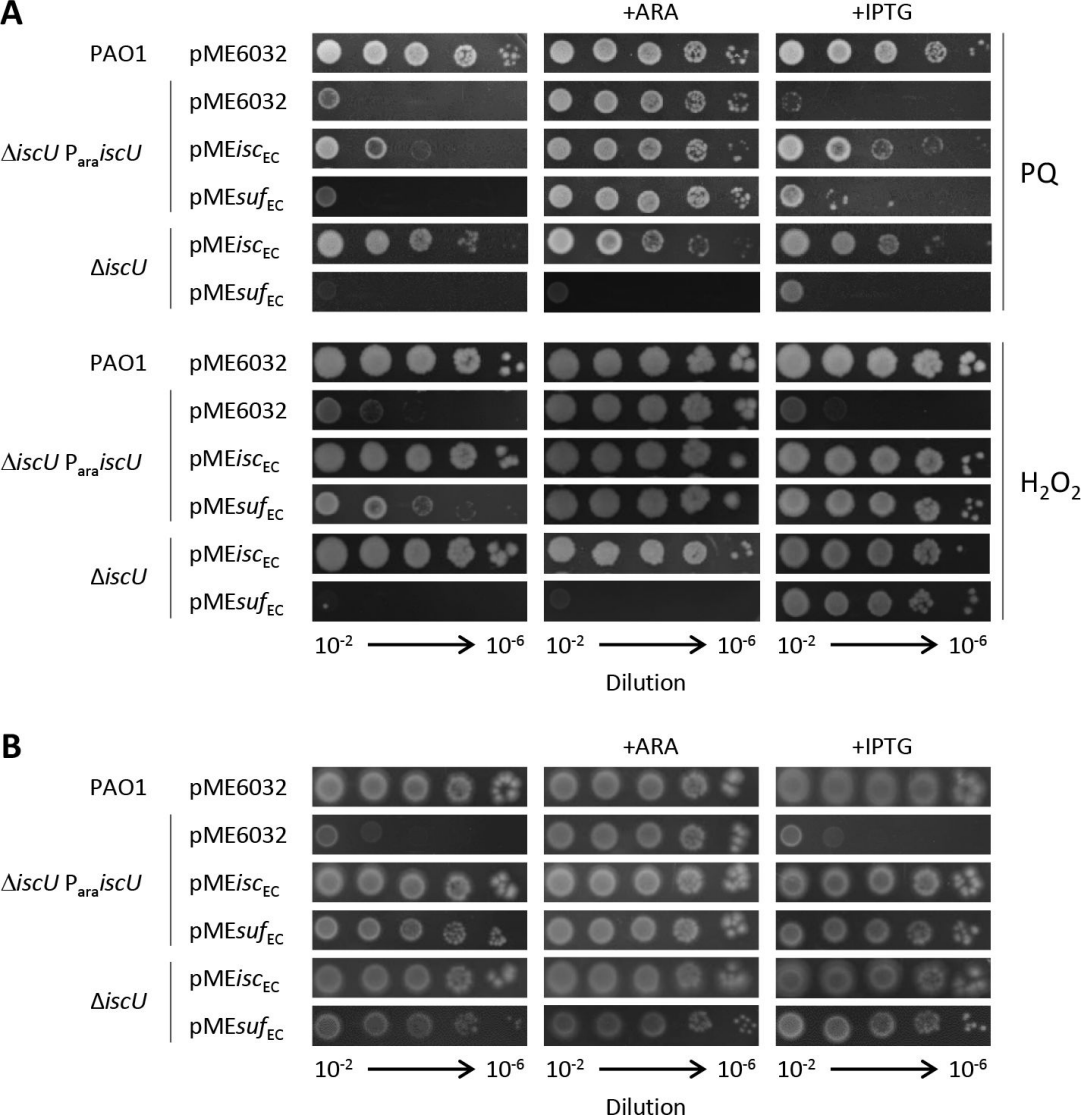
Effect of *E. coli* ISC and SUF systems on *P. aeruginosa* oxidative stress resistance and anaerobiosis. (**A**) Colony growth of the *P. aeruginosa* Δ*iscU* P_ara_*iscU* conditional mutant or the Δ*iscU* deletion mutant carrying the plasmid pME6032 with the entire *isc* operon (*isc*_EC_) or the entire *suf* operon (*suf*_EC_) of *E. coli* under an IPTG-inducible promoter on MH agar plates supplemented with H_2_O_2_ or paraquat (PQ) at 0.25 × MIC for the wild type (corresponding to 0.25 mM and 0.125 mM, respectively). (**B**) Colony growth of the strains described in panel A grown under anaerobiosis conditions. PAO1 and Δ*iscU* P_ara_*iscU* with the empty plasmid pME6032 were used as positive and negative controls, respectively. When indicated, arabinose (ARA) and IPTG were added at 0.5% and 0.5 mM, respectively. Images are representative of three independent assays.

Since in *E. coli* SUF is thought to be the stress responding system, we investigated whether SUF-mediated [Fe-S] cluster biogenesis in *P. aeruginosa* had any positive effect on oxidative stress resistance. The *P. aeruginosa* Δ*iscU* P_ara_*iscU* conditional mutant and the ∆*iscU* strain carrying either pME*isc*_EC_ or pME*suf*_EC_ were grown in the presence of hydrogen peroxide (H_2_O_2_) or paraquat (PQ), a superoxide generating compound, at sub-MIC concentrations. Expression of the *E. coli* ISC system allowed growth of *P. aeruginosa iscU* mutants both in the presence of PQ and H_2_O_2_ (Fig. 4A) even if rescue was less efficient on PQ-containing plates than on H_2_O_2_. This indicated that *E. coli* ISC is capable of sustaining ROS challenge when expressed in *P. aeruginosa*. Surprisingly, *E. coli* SUF expression largely failed to rescue IscU-depleted *P. aeruginosa* cells on PQ-containing plates, while it was able to sustain growth on H_2_O_2_ containing plates (Fig. 4A; Fig. S5).

**Fig 5:**
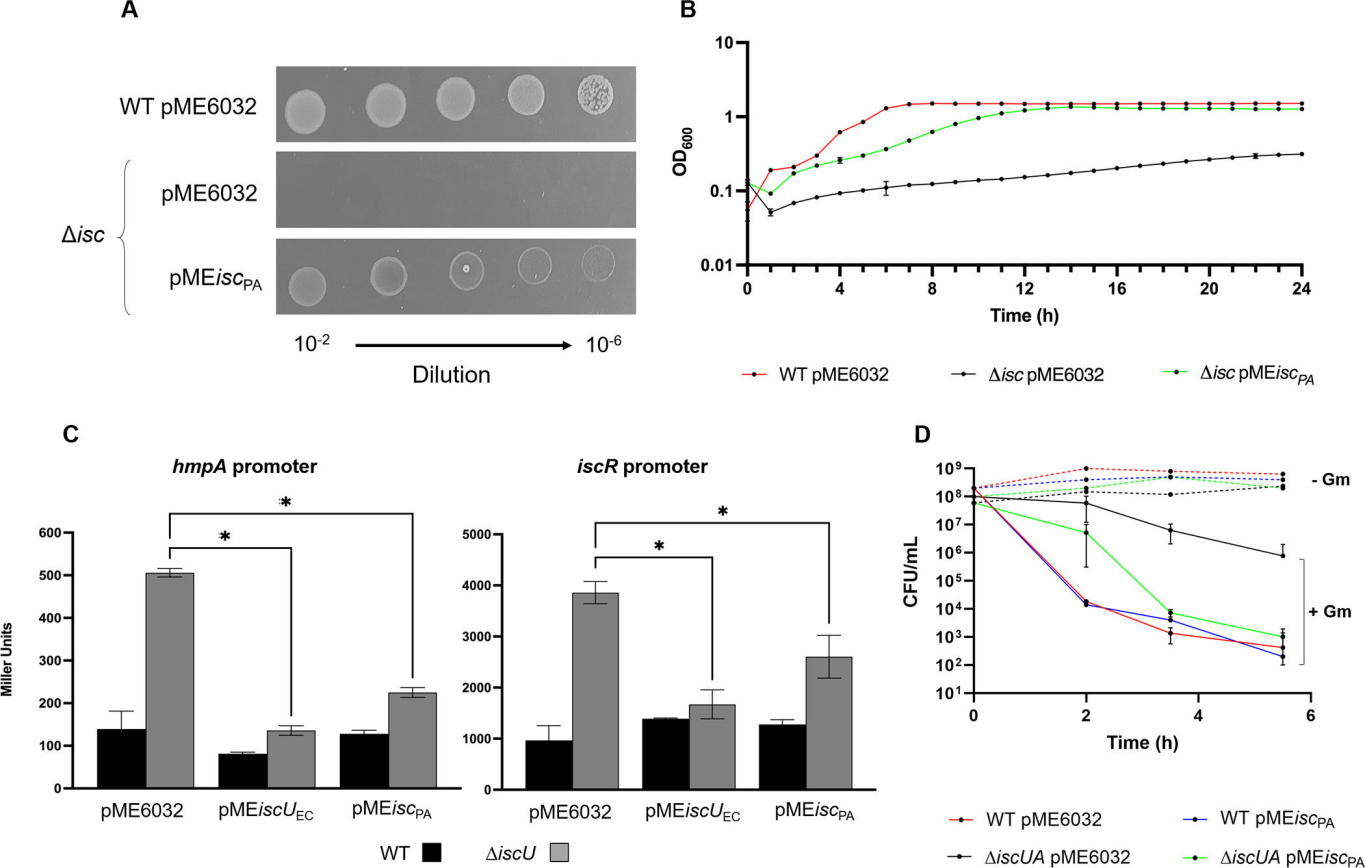
*P. aeruginosa* ISC system can replace the endogenous ISC system in *E. coli*. (**A**) Colony growth of the *E. coli* wild type (WT) and Δ*isc* mutant, carrying the empty plasmid pME6032 or the one with the entire *isc* operon from *P. aeruginosa* (pME*isc*_PA_), on M9 glucose agar plates. (**B**) Planktonic growth of *E. coli* WT and Δ*isc* strains carrying pME*isc*_PA_ or the empty plasmid at 37°C in M9 glucose. (**C**) β-Galactosidase activity of the *E. coli* WT (black bars) and Δ*iscU* (gray bars) strains carrying the chromosomal fusions P*hmpA::lacZ* (left panel) or P*iscR::lacZ* (right panel) and the empty plasmid pME6032 or pME*isc*_PA_ cultured in LB. The plasmid containing the *E. coli iscU* (pME*iscU*_EC_) was used as control. β-Galactosidase activity is expressed as Miller units. The asterisks indicate statistically significant differences (*P* < 0.05) with respect to the Δ*iscU* mutants carrying the empty plasmid. (**D**) Survival curves of WT and Δ*iscUA* mutant carrying the empty plasmid pME6032 or pME*isc*_PA_, exposed to 5 µg/mL gentamycin (Gm) in LB medium. The dotted lines represent the survival of the same strains without Gm addiction. For all the experiments, IPTG was added at the concentration of 100 µM. Data are the mean (±SD) or are representative of at least three independent assays.

We also compared the growth complementing activity of *E. coli* SUF and ISC systems in IscU-depleted *P. aeruginosa* under anaerobiosis, which is expected to reduce endogenous ROS formation (31, 32). Anaerobic growth of IscU-depleted or Δ*iscU P. aeruginosa* cells was found to be equally promoted by the expression of either *E. coli* ISC or SUF (Fig. 4B). Interestingly, under anaerobic conditions, the growth of ISC-defective *P. aeruginosa* mutants carrying the pME*suf*_EC_ construct was almost fully restored even in the absence of IPTG (Fig. 4B) in contrast to what was observed in aerobiosis (Fig. 3A; Fig. S3). Western blot analysis showed that IPTG-inducible expression from pME6032 derivatives is similar in both aerobic and anaerobic conditions (Fig. S4).

Altogether these results revealed that *E. coli* SUF can substitute for the endogenous ISC system of *P. aeruginosa* in promoting [Fe-S] cluster biogenesis and growth. A surprising observation though is that SUF appears to be less efficient for helping *P. aeruginosa* to cope with ROS than the ISC system of either *P. aeruginosa* or *E. coli*.

### *P. aeruginosa* ISC can replace the *E. coli* ISC system

Results above demonstrated that ISC of *E. coli* can substitute for ISC of *P. aeruginosa*. Conversely, we tested whether the *P. aeruginosa* ISC can replace the *E. coli* endogenous ISC. To do this, the entire *isc* operon (*iscSUAhscBAfdx*) of *P. aeruginosa* was cloned in the pME6032 vector, generating the pME*isc*_PA_ plasmid, which was introduced into an *E. coli* ∆*isc* strain lacking the genes *iscSUAhscBAfdx* (Table S1). The *E. coli* Δ*isc* mutant grows very poorly in M9 minimal medium because of the inefficient functioning of several [Fe-S] cluster proteins required for synthesizing amino acids, vitamins, and cofactors (33). As a side comment, this implies a poor efficiency of the SUF system in maturing the set of [Fe-S] proteins involved in these biosynthetic processes. Our results showed that the ISC system of *P. aeruginosa* rescued the growth defect of the *E. coli* ∆*isc* strain both in liquid and solid minimal medium M9 (Fig. 5A and B).

Next, we tested the capacity of *P. aeruginosa* ISC to support the activity of two *E. coli* [Fe-S] cluster-containing transcriptional regulators, IscR, repressor of the *isc* operon, and NsrR, repressor of several genes among which *hmpA*, involved in cell protection against nitric oxide (34). Assays were performed in the rich medium LB. In the *E. coli* Δ*iscU* mutant carrying the pME6032 plasmid, a fourfold increase in promoter activity with respect of the corresponding wild-type control was observed (Fig. 5C). This activity was due to alleviation of the repression exerted by IscR and NsrR on *iscR* and *hmpA* promoters, respectively. Expression of the ISC system of *P. aeruginosa* caused a decrease in the activity of both *iscR* and *hmpA* promoters (Fig. 5C), indicating maturation of IscR and NsrR. Finally, we tested the ability of *P. aeruginosa* ISC to efficiently mature the *E. coli* respiratory complexes I and II. As described above, *E. coli* lacking a functional ISC system exhibits an increased tolerance to aminoglycoside due to inefficient maturation of both complexes and associated decrease in pmf level (12). Therefore, gentamycin killing assays were run with *E. coli* wild type and Δ*iscUA* strains expressing or not the *P. aeruginosa* ISC system. While the Δ*iscUA* carrying the empty plasmid displayed tolerance to gentamycin, expression of *P. aeruginosa* ISC was able to restore susceptibility to antibiotics at levels comparable to the wild-type strain (Fig. 5D). These results showed that *E. coli* is equally efficient at making [Fe-S] clusters with either the endogenous or the *P. aeruginosa* ISC system.

### *E. coli* lacking both ISC and SUF but using the *P. aeruginosa* ISC system is viable

**Fig 6:**
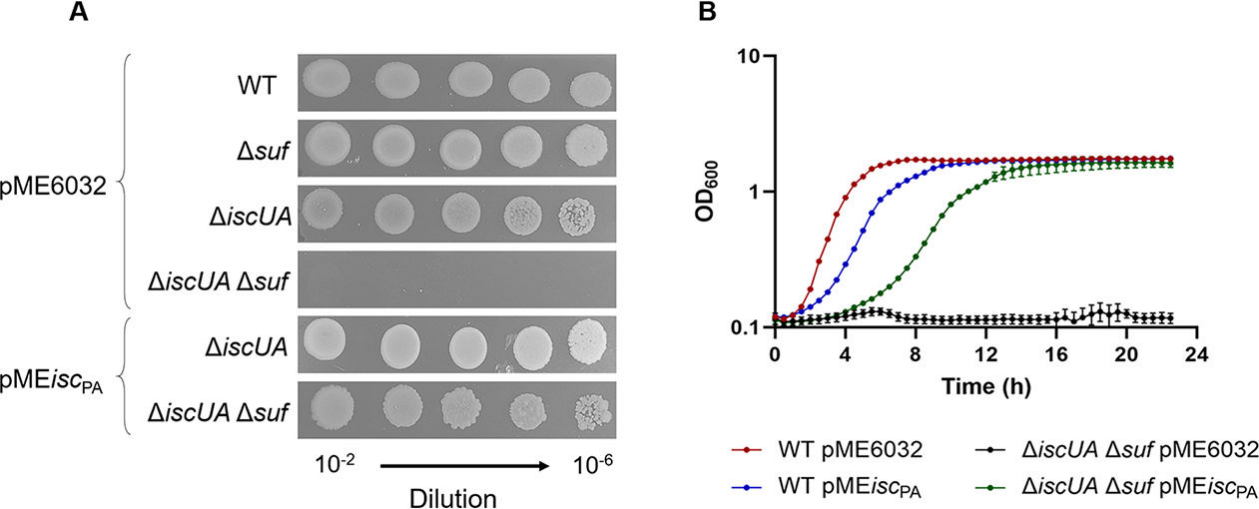
*P. aeruginosa* ISC functionally replaces *E. coli* ISC and SUF systems. (**A**) Colony growth of the *E. coli* wild type (WT) and Δ*iscUA* Δ*suf* strains carrying the plasmid pME6032 empty or containing the entire *isc* operon from *P. aeruginosa* (pME*isc*_PA_), on LB agar plates. The single mutants Δ*iscUA* Δ*suf* carrying pME6032 were used as controls. (**B**) Planktonic growth of *E. coli* WT and Δ*iscUA* Δ*suf* strains carrying pME*isc*_PA_ or the empty plasmid at 37°C in LB. For all the experiments, IPTG was added at the concentration of 100 µM. Data are the mean (±SD) or are representative of at least three independent assays.

We tested whether the ISC system of *P. aeruginosa* would be able to substitute for the lack of both ISC and SUF systems in *E. coli*. To assess this, the pME*isc*_PA_ plasmid was inserted into the *E. coli* ∆*iscUA* ∆*suf* double mutant. First, colony growth assays were used to test the ability of pME*isc*_PA_ to sustain the growth of the non-viable *E. coli* double-mutant Δ*iscUA*Δ*suf* on a rich medium under aerobic conditions. Note that this mutant can be cultivated if grown in the presence of mevalonate (MVA) and arabinose as it contains the genes for the [Fe-S]-independent eukaryotic isoprenoid biosynthesis pathway under an arabinose-dependent promoter (35). This mutant, transformed with pME6032 empty plasmid, was not able to grow in LB supplemented with IPTG (Fig. 6A). Interestingly, the growth of the Δ*iscUA*Δ*suf* mutant was restored when transformed with pME*isc*_PA_, showing the ability of *P. aeruginosa* ISC to support [Fe-S] cluster biogenesis in *E. coli* lacking functional endogenous systems. The same observation was obtained when performing growth assays in a liquid medium, as the ∆*iscUA* ∆*suf E. coli* strain could grow solely if expressing the *P. aeruginosa* ISC system (Fig. 6B). These results demonstrated that, when expressed in *E. coli*, the *P. aeruginosa* ISC system is able to mature all *E. coli* [Fe-S] cluster proteins required to grow in the conditions used, in particular IspG and IspH, the two [Fe-S] cluster proteins required for synthesizing isoprenoids that are precursors for peptidoglycan biosynthesis and essential for growth.

### *P. aeruginosa* ISC is not able to replace the *E. coli* SUF under stress conditions

**Fig 7:**
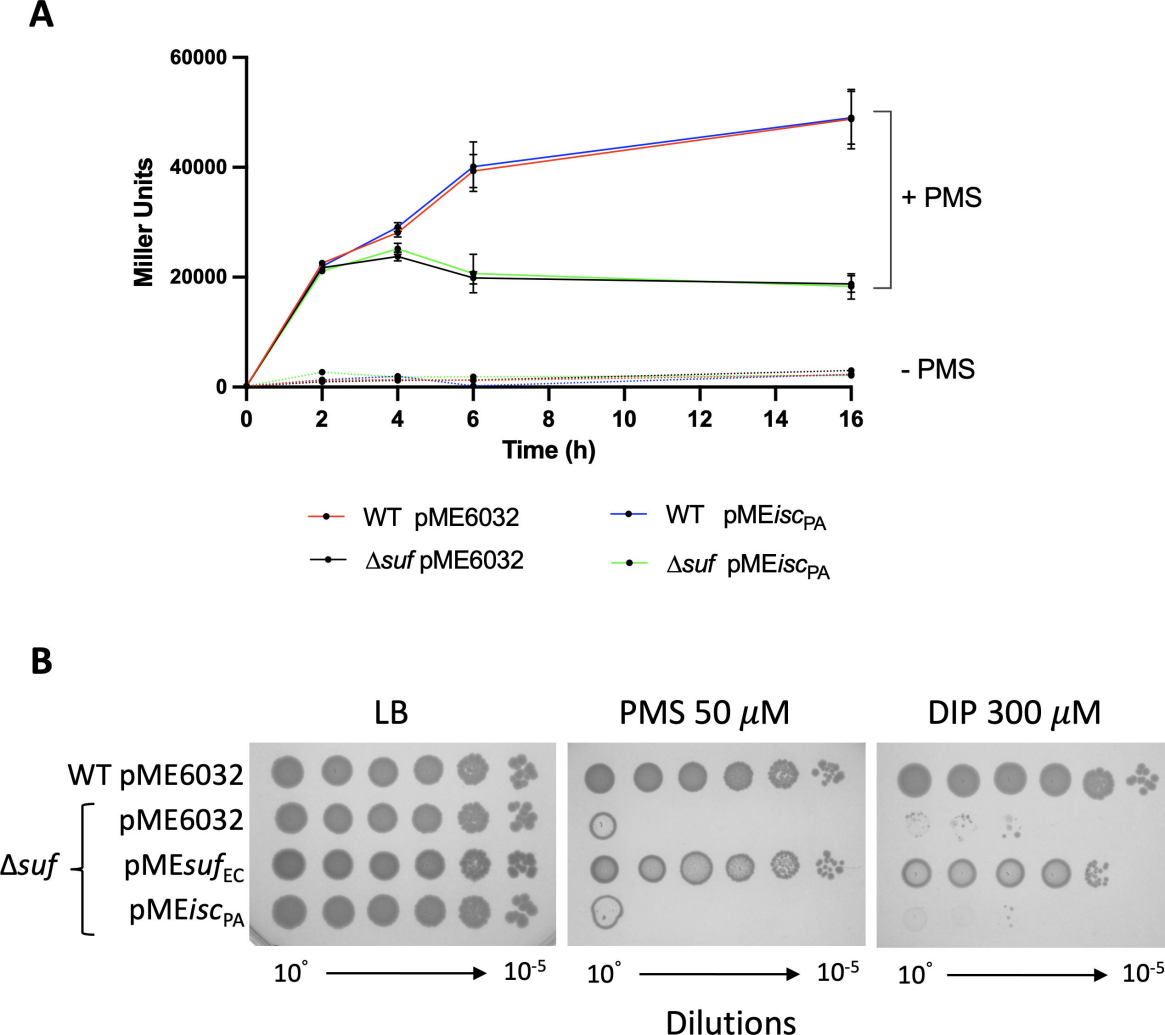
*P. aeruginosa* ISC is not able to replace the *E. coli* SUF under oxidative stress conditions. (**A**) The *E. coli* wild type (WT) and Δ*suf* strains carrying the chromosomal fusion P*soxS::lacZ* were transformed with the empty plasmid pME6032 or pME*isc*_PA_. At time zero, phenazine methosulfate (PMS, 30 µM) was added, and β-galactosidase activity was monitored at the indicated time points and expressed as Miller units. The dotted lines represent β-galactosidase activity of the same strains without PMS addiction. (**B**) Colony growth on LB agar, supplemented or not with PMS (50 µM) or dipyridyl (DIP, 300 µM), of the *E. coli* Δ*suf* mutant strain carrying the empty plasmid pME6032 or pME*isc*_PA_. WT or Δ*suf* transformed with the plasmid carrying the *suf* operon of *E. coli* (pME*suf*_EC_) were used as controls. For all the experiments, IPTG was added at the concentration of 100 µM. Data are the mean (±SD) or are representative of at least three independent assays.

Next, we tested whether *P. aeruginosa* ISC would be able to substitute for SUF when *E. coli* is exposed to ROS. We first measured the activity of the transcriptional regulator SoxR, an [Fe-S] cluster-dependent transcriptional activator responding to redox cycling drugs. Previous work showed that, under exposure to the ROS activator phenazine methosulfate (PMS), only SUF can mature SoxR and permit expression of P*soxS::lacZ* transcriptional fusion target (13). Fig. 7A shows that P*soxS* activity is similar in the wild type and Δ*suf* backgrounds for the first 2 h of growth (because ISC is still active) (13), but after 3 h, the activity decreases as SoxR loses its cluster since ISC is not efficient anymore and SUF is missing (Fig. 7A). Interestingly, in the ∆*suf* strain expressing the *P. aeruginosa* ISC system, no P*soxS::lacZ* associated activity was observed either, indicating that the *P. aeruginosa* ISC system was unable to deliver [Fe-S] cluster onto SoxR under PMS-induced oxidative stress (Fig. 7A). To further explore this issue, we tested the capacity of the *P. aeruginosa* ISC system to rescue the growth of the *E. coli* ∆*suf* strain under both oxidative stress and iron starvation. *P. aeruginosa* ISC was unable to support growth of the ∆*suf* strain on plates supplemented with PMS or the iron chelator dipyridyl (DIP) (Fig. 7B). These results strongly support the view that *P. aeruginosa* ISC is not efficient in sustaining [Fe-S] cluster biogenesis under stress conditions in *E. coli*.

### Comparison of *E. coli* and *P. aeruginosa* abilities to sustain stress

**Fig 8:**
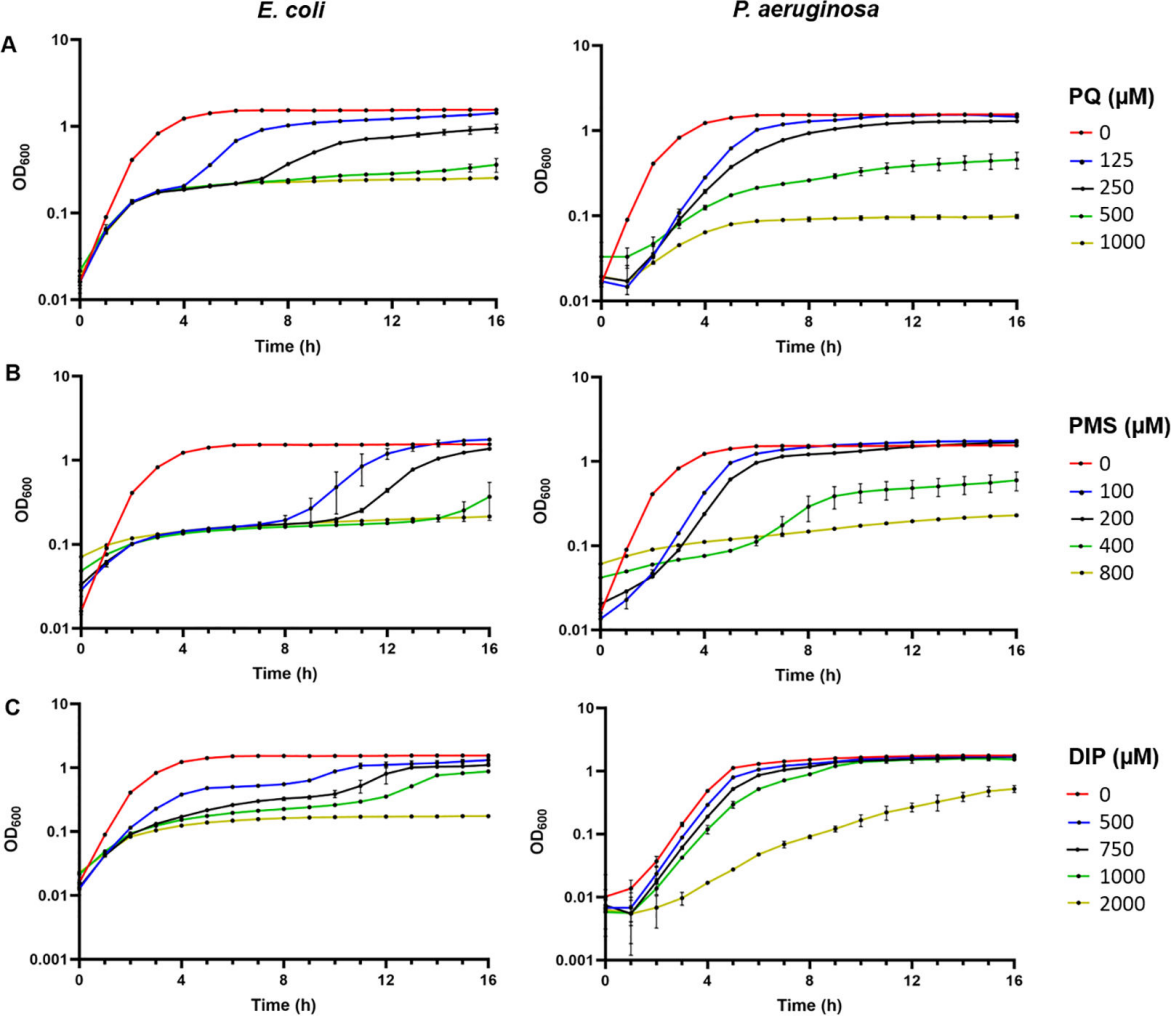
Comparison of *E. coli* and *P. aeruginosa* abilities to sustain stress. Planktonic growth of the *E. coli* MG1655 (left graphs) and *P. aeruginosa* PAO1 (right graphs) strains in LB supplemented or not with the concentrations indicated in the graphs (expressed in μM) of (**A**) PQ, (**B**) PMS, and (**C**) DIP. Data are the mean (±SD) of at least three independent assays.

The above results point to differential capacities of ISC and SUF systems to operate under stress conditions. The fact that *P. aeruginosa* does not possess the stress-responding SUF system led us to compare capacities of both strains to thrive in the presence of stressors targeting [Fe-S] clusters, i.e., PMS, PQ, H_2_O_2_, and DIP. Comparison of the growth of *E. coli* MG1655 and *P. aeruginosa* PAO1 wild-type strains in the presence of varying concentrations of stressors revealed unexpected differences, pointing to an overall higher resistance of *P. aeruginosa* PAO1 than *E. coli* MG1655. At 125 and 250 µM PQ, *E. coli* exhibited a marked delay in resuming growth upon inoculation, which was only marginal for *P. aeruginosa*; moreover, at 500 µM PQ, *E. coli* growth ceased, whereas *P. aeruginosa* growth slowed down but remained effective (Fig. 8A). *E. coli* showed a drastic increased time period before to resume growth at 100 µM PMS and did not grow at 400 µM PMS, whereas *P. aeruginosa* was modestly altered until 200 µM PMS and exhibited some residual growth also at 400 µM PMS (Fig. 8B). Regarding resistance to iron depletion, *E. coli* showed atypical multiphasic growth profiles as well as extended lag phases from 500 µM DIP, whereas *P. aeruginosa* was almost not affected until 2 mM DIP (Fig. 8C). In contrast, PAO1 showed delayed growth in the presence of all H_2_O_2_ concentrations tested that was not observed for MG1655 (Fig. S6). Thus, overall *P. aeruginosa* showed a much better capacity to resist to challenges imposed by chemicals predicted to lead to superoxide increase (PMS, PQ) and iron limitation (DIP).

### Distribution of ISC, SUF, and NIF systems in *Pseudomonadales*

**Fig 9:**
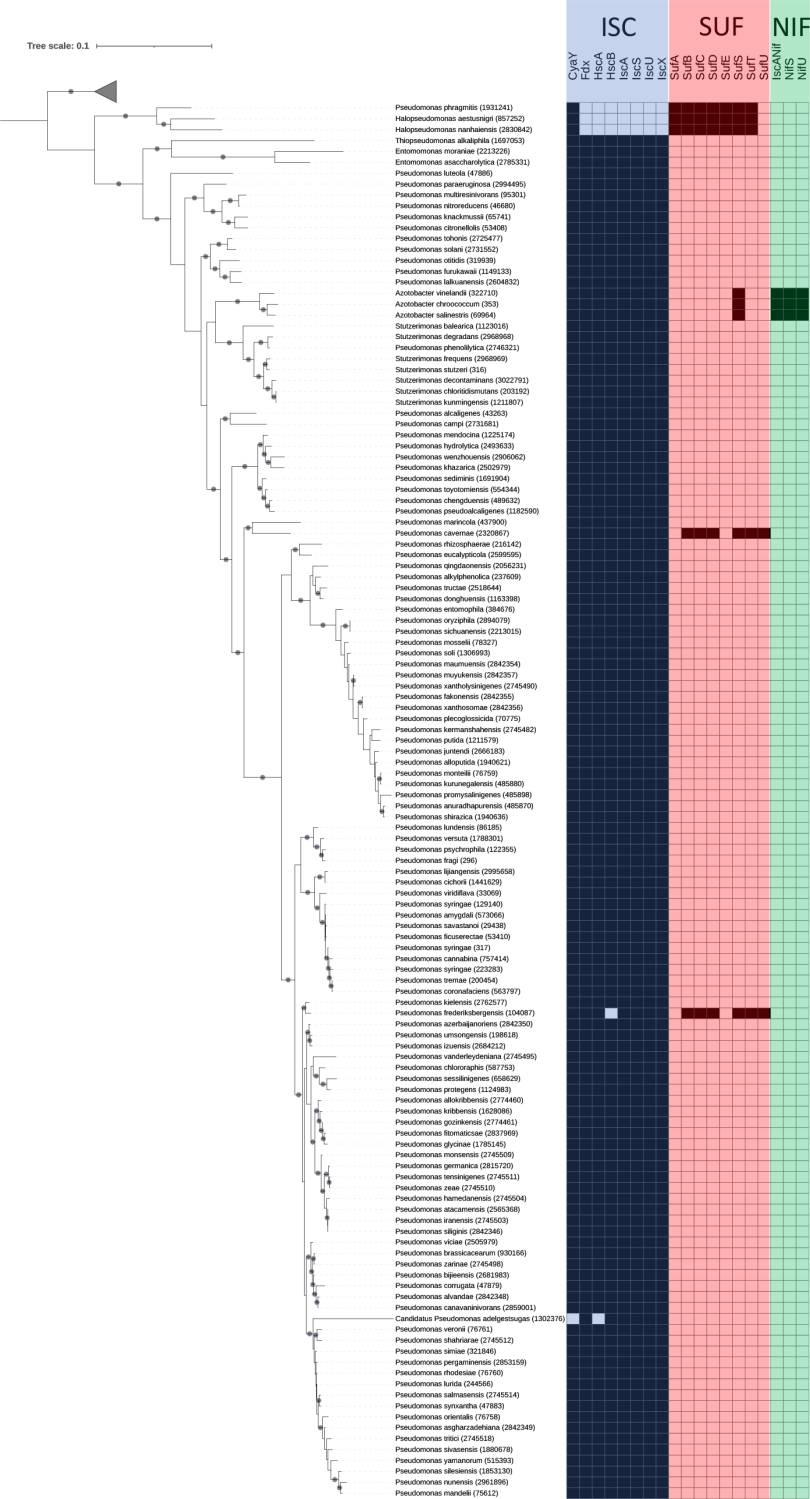
Taxonomic distribution of ISC, SUF, and NIF components in *Pseudomonadales*. The reference tree has been inferred using a concatenation of If2, RpoB, and RpoC (IQ-TREE, LG + R10, 3,573 amino-acid positions, 201 sequences). The group of *Oceanospirillales* has been collapsed and used as an outgroup (represented as a gray triangle). The dots on branches indicate an ultrafast bootstrap value ≥0.95. The scale bar indicates the average number of substitutions per site. The numbers after species name correspond to the NCBI taxonomic ID. The presence of proteins in proteomes is indicated by dark-colored squares.

Our recent thorough analysis of over 9,000 bacterial genomes revealed that the vast majority of bacteria had only one [Fe-S] cluster biogenesis system and that it was most of the time SUF rather than ISC (5). Therefore, we wondered how SUF, ISC, and NIF were distributed specifically in the *Pseudomonadales* clade. For this, we searched for the homologs of components of [Fe-S] cluster biosynthesis machineries in 139 genomes of *Pseudomonadales* and mapped their presence/absence on the associated species tree (Fig. 9). Concerning the species tree, we observed that *Pseudomonadales* are not forming a monophyletic group, *Marinobacter*, *Pseudohongiella*, *Permianibacter*, and *Ketobacter* being mixed to *Oceanospirillales* (in the outgroup, see Supplementary data). Furthermore, *Pseudomonas* are also polyphyletic, with *Entomomonas*, *Azotobacter*, *Stutzerimonas* branching within *Pseudomonas*. The large majority of *Pseudomonadales* possesses an ISC system, with the exception of the basal lineages (*P. phragmitis* and *Halopseudomonas*) that have only one SUF similar to the *E. coli* type (SufABCDSET). A few organisms have an additional system: *Azotobacter* possess the NIF system that has been extensively studied in *A. vinelandii* (6–8) and two *Pseudomonas* species (*P. cavernae* and *P. frederiksbergensis*) have a SUF system that is similar to SUF from *Terrabacteria*, i.e., SufBCDSTU (5). This type of SUF has been likely acquired by horizontal gene transfer from this group. Altogether, this analysis reveals the heterogeneity of the *Pseudomonadales* clade, with all except three *Pseudomonas* species having selected ISC over SUF.

## DISCUSSION

[Fe-S] clusters are essential and [Fe-S] cluster biogenesis machineries ISC and SUF have been the focus of multiple investigations in the *E. coli* bacterium model. Principles derived from these studies are that ISC and SUF share targets but operate under different conditions such as to insure a continuum synthesis of [Fe-S] clusters across a wide variety of conditions. In this regard, the human pathogen *P. aeruginosa* deserves specific attention for two reasons. First, studies in *E. coli* indicated that SUF is a better fit for oxidative stress and iron limiting conditions, as met by *P. aeruginosa* during its multiplication in its hosts. Yet, *P. aeruginosa* has only ISC. Second, a recent thorough phylogenomic investigation revealed that the vast majority of bacteria have only one [Fe-S] cluster biogenesis machinery that, in most cases, is SUF (5), again in contradiction with the situation observed in *P. aeruginosa*.

Here, we observed that the *E. coli* and *P. aeruginosa* ISC were fully exchangeable, i.e., both a *P. aeruginosa* (resp. *E. coli*) lacking its own ISC but expressing the *E. coli* (resp. *P. aeruginosa*) ISC exhibited wild type like physiological features. Such interspecies complementation is consistent with the molecular conservation of ISC systems and demonstrates that ISC can catalyze [Fe-S] cluster biogenesis in phylogenetically distant genetic backgrounds. Importantly, *P. aeruginosa* lacking endogenous ISC but expressing *E. coli* ISC showed resistance to stressors such as H_2_O_2_ and, to some extent, PQ. However, we noticed that overproduction of ISC was necessary for such complementation, yet this definitively demonstrated that both systems are exchangeable under both normal and stress conditions.

*P. aeruginosa* has no stress responding SUF and, in *E. coli*, ISC and SUF are thought to be redundant in maturating the same set of cellular targets, though in different conditions. The question then arose of how *P. aeruginosa* would grow if relying on a SUF, rather than an ISC system. From the redundancy observed between ISC and SUF in *E. coli*, a possible expectation was that SUF would be able to substitute for ISC in *P. aeruginosa*. This prediction was met according to several biochemical, physiological, and genetic readouts, showing that ISC-deficient *P. aeruginosa* expressing *E. coli* SUF is viable and almost behaves like the wild type under normal conditions. From the alleged intrinsic stress-resistance character of SUF in *E. coli*, one would predict that SUF might endow *P. aeruginosa* with an enhanced capacity to handle oxidative stress. This prediction fell short, as *E. coli* SUF was unable to complement ISC-deficient *P. aeruginosa* for PQ sensitivity and overproduction of SUF was necessary for reaching full complementation for H_2_O_2_ resistance. Thus, these experiments showed that *P. aeruginosa* making [Fe-S] clusters with SUF might be less efficient in adapting to stress than when making them with ISC, providing an experimental rationale for the evidence that *P. aeruginosa* strains, and as a matter of fact most *Pseudomonas* species, retained ISC over SUF. Moreover, this suggested that the enhanced resistance, which SUF provides *E. coli* with, is not only due to intrinsic features of the SUF machinery but has to be appreciated within the context of the cellular system. Presumably, expression and maturation of heterologous protein complexes, interactions with other [Fe-S] cluster biogenesis factors acting downstream the SUF system, and intrinsic features of targeted substrates (such as [Fe-S] cluster stability) have to be put into the equation to fully predict outcome of [Fe-S] cluster-dependent phenotype under stress conditions. Interestingly, we found that *E. coli* SUF was much more efficient in complementing growth of ISC-deficient *P. aeruginosa* under anaerobic conditions. This suggests that *P. aeruginosa* may have a lower demand for [Fe-S] clusters when growing anaerobically or that the *E. coli* SUF system works better under low oxygen conditions in the *P. aeruginosa* cellular context.

The necessity to consider the whole cellular system is, furthermore, indicated by our experiments in *E. coli* as the host. We observed that the *P. aeruginosa* ISC system was able to provide ∆*iscUA* ∆*suf E. coli* with capacity to grow under normal conditions. In essence, this is confirmatory of the capacity of ISC systems to be exchanged, as under balanced growth conditions only ISC is required. In contrast, under oxidative stress conditions, wherein the SUF system is used by *E. coli*, the *P. aeruginosa* ISC system was unable to rescue *E. coli* lacking both ISC and SUF. This means that while when operating in its native cellular context, the ISC system of *P. aeruginosa* allows growth under stress conditions, it is unable to bring in along this “stress resistance” capacity into the *E. coli* context. This is perfectly symmetric to the fact that whereas in its “native” cellular context, the *E. coli* SUF system is required for “stress resistance,” it is unable to transfer this trait to *P. aeruginosa*. This indicates that the contribution of [Fe-S] cluster biogenesis system is only one of the many stress adaptation systems used by bacteria. As a matter of fact, a simple comparison of *E. coli* and *P. aeruginosa* capacities to sustain ROS and iron limitation challenges revealed how the latter is better armed than the former. Presumably in *Pseudomonas*, the potential weakness in having one single ISC system has been compensated by acquiring other adaptation devices such as ROS-detoxifying enzymes and siderophores to help the bacterium resist to ROS and iron limitation. It is tempting to hypothesize that *P. aeruginosa* may have evolved resistance to redox agents also because it must tolerate the pyocyanin it makes. We previously speculated on the structural, and possibly functional, relatedness between redox cycling drugs and pyocyanin export system (13).

Since many [Fe-S] cluster-containing proteins play important roles in bacterial metabolism, stress response, and resistance, [Fe-S] cluster biogenesis systems are expected to be essential for growth and pathogenicity and, therefore, could represent exploitable targets for the development of new antibacterial. As expected here, we demonstrated that the ISC system is essential in the pathogen *P. aeruginosa*. This is consistent with several independent transposon mutagenesis projects that generally failed to obtain *P. aeruginosa* transposon mutants in *isc* genes (36–39). While this evidence supports the notion that cluster biogenesis is a potential target for antibacterial drug discovery, there are at least two important issues that could potentially hinder the development of antipseudomonal drugs targeting [Fe-S] cluster biogenesis. First, a homolog of the bacterial ISC system is also present in mitochondria (40). This implies that any potential ISC inhibitor should be accurately evaluated and developed to avoid or reduce the probable inherent toxicity toward eukaryotic cells. Moreover, in this study, we have observed that IscU depletion, which should mirror the effect of ISC inhibition, blocks bacterial growth without killing the cells and makes *P. aeruginosa* cells more tolerant to bactericidal antibiotics, likely due to a general inhibitory effect on bacterial metabolism. If this were also true for ISC inhibitors, such compounds would not represent a suitable therapeutic strategy for immunocompromised patients, whose immune system is not able to clear the infection on its own. These findings highlight the requirement for exhaustive genetic-phenotypic analysis of a putative antibacterial target before to embark on further dedicated strategies.

## MATERIALS AND METHODS

### Bacterial strains and growth media

Bacterial strains and plasmids used in this study are listed in Table S1. Bacteria were cultured in Lysogeny Broth, Lennox formulation (LB; Acumedia) for genetic manipulation. Growth assays were performed in Mueller-Hinton broth (MH; Difco), LB or M9 supplemented with glucose (0.4%), CaCl_2_ (100 µM), and MgSO_4_ (1 mM). Solid media contained 1.5% agar. Biofilm assays were performed in MH or Tryptic soy broth (TSB) as indicated. When specified, growth media were supplemented with arabinose, isopropyl-β-d-thiogalactopyranoside (IPTG), hydrogen peroxide (H_2_O_2_), phenazine methosulfate (PMS), dipyridyl (DIP), and/or paraquat (PQ) at the indicated concentrations. Mevalonate (MVA) at 0.5 mM was added when indicated. When required, antibiotics were added at the following concentration for *E. coli* (the concentration used for *P. aeruginosa* are shown between brackets): ampicillin 100 µg/mL, kanamycin 50 µg/mL, nalidixic acid 20 µg/mL, gentamycin 5 µg/mL, chloramphenicol 30 µg/mL (350 µg/mL), tetracycline 12 µg/mL (50–100 µg/mL). Mevalolactone (MVL) was purchased from Sigma-Aldrich and resuspended in H_2_O at final concentrations of 1 M. To prepare MVA, an equal volume of 1 M KOH was added to 1 M MVL and incubated at 37°C for 30 min.

### Growth assays

Growth assays in liquid media were performed in 96-well microtiter plates (200 µL of total volume in each well) or in flasks at 37°C and vigorous shaking (200 rpm). Growth was measured as the optical density at 600 nm (OD_600_) of bacterial cultures in a Tecan Spark 10 M microtiter plate reader for microtiter plates or of appropriate dilutions in a sterile growth medium in a spectrophotometer for flask cultures. To obtain IscU-depleted *P. aeruginosa* cells for further analyses, a previously described dual-refresh culturing strategy in flasks was used (41). Briefly, cells were cultured overnight in the presence of 0.5% arabinose and then refreshed at high cell density (1:20 dilution) in the absence of arabinose, or in the presence of 0.5% arabinose as control, cultured for 2 h and then refreshed again (1:30 dilution) in the same medium. IscU-depleted cells were collected as soon as a growth defect was observed with respect to control cultures. For experiments with the *E. coli* double-mutant Δ*iscUA* Δ*suf*, overnight cultures were prepared in the presence of 0.5 mM of MVA in the medium and then refreshed in the absence of MVA, or in the presence of 0.5 mM of MVA as control. For growth in M9, overnight cultures were prepared in LB and then refreshed in M9 Glucose. Growth assays on solid media were performed by spotting 5 µL of serial 10-fold dilutions from bacterial suspensions normalized in saline at an OD_600_ = 1, from late-exponential cultures grown in the presence or in the absence of compounds indicated in the text, in MH, LB, or M9 glucose, as reported in the text. For anaerobic growth, agar plates were supplemented with 0.1% NaNO_3_ and incubated in a GENBox Jar 2.5 L in the presence of a GENbox anaerobic generator (bioMérieux, Marcy l'Etoile, France).

### Generation of plasmids and recombinant strains

Recombinant DNA procedures have been described elsewhere (42). All DNA fragments for cloning were amplified by PCR with Pfu (Promega) or Q5 Hot Start High-Fidelity (New England Biolabs) DNA Polymerases, using the genomic DNA of *P. aeruginosa* PAO1 or of *E. coli* MG1655 as the template. Primers and restriction enzymes used for cloning are described in Table S2. All constructs generated in this study were verified by restriction analysis and DNA sequencing.

The integration-proficient construct mini-CTX1-*araC*P_araBAD_*iscU*_PA_ was generated by replacing the *tolB* gene in mini-CTX1-*araC*P_BAD_*tolB* (41) with the coding sequence of the *iscU*_PA_ gene. In this construct, the *iscU*_PA_ coding sequence is cloned downstream of an arabinose-dependent regulatory element *araC*-P_araBAD_ optimized for *P. aeruginosa* by the modification of the ribosome-binding site (43). The deletion mutagenesis construct pDM4Δ*iscU*_PA_ was obtained by directionally cloning two DNA regions upstream and downstream of the *iscU*_PA_ coding sequence into pBluescript II KS+ (Stratagene), followed by DNA sequencing and subcloning of the entire insert encompassing the *iscU*_PA_ upstream and downstream regions into the suicide vector pDM4 (44).

The *P. aeruginosa* PAO1 Δ*iscU* P_ara_*iscU* conditional mutant was generated using a previously described strategy (41). Briefly, the *iscU*_PA_ coding sequence under the control of modified arabinose-dependent regulatory element *araC*-P_BAD_ (see above) was integrated into the *attB* neutral site of the *P. aeruginosa* PAO1 chromosome, and excision of the mini-CTX1 plasmid backbone was obtained by Flp-mediated recombination as described (45). In-frame deletion of the endogenous copy of *iscU*_PA_ was obtained under permissive condition (i.e., growth in the presence of 0.5% arabinose) using the *sacB*-based suicide construct pDM4Δ *iscU*_PA_ as previously described (46).

The expressing constructs pME*iscU*_EC_, pME*isc*_EC_, pME*suf*_EC_ and pME*sufBCD*_EC_, and pME*isc*_PA_ (Table S1) were generated by directionally cloning the PCR-amplified gene(s) or operon of interest, without the endogenous promoter, into the IPTG-inducible shuttle vector pME6032 (47), downstream of the P*tac* promoter. The pME6032 derivatives were introduced into *P. aeruginosa* or *E. coli* strains by transformation using chemically competent cells.

### Biofilm assays

Biofilm assays were performed as previously described (48), with few modifications. For the microtiter plate biofilm assay, *P. aeruginosa* strains were cultured overnight in MH supplemented with 0.5% arabinose, refreshed at an OD_600_ = 0.002 in fresh MH containing or not 0.5% arabinose, and dispensed in 96-well polystyrene plates (150 µL per well). After 24 h incubation at 37°C under static conditions, the wells were washed several times with distilled water. Attached cells were stained with 0.1% crystal violet (175 µL) at room temperature (RT) for 15 min and washed several times with distilled water to remove unbound dye. Biofilm-bound crystal violet was eluted in absolute ethanol (200 µL) at RT for 15 min, and for each well, 100 µL of the resulting solution was aliquoted in a sterile microtiter plate. The released crystal violet was measured as OD_600_ in a Tecan Spark 10M microtiter plate reader. Triplicate independent experiments with at least three wells per condition were performed.

For the flow-cell biofilm assay, biofilm flow chambers were inoculated with overnight cultures in TSB containing 0.5% arabinose of *P. aeruginosa* strains constitutively expressing GFP from pMRP9-1 (Table S1), which were diluted at an OD_600_ of 0.15 in 1% TSB. A flow of 1% TSB (supplemented with 0.5% arabinose when required) was initiated after 2 h using a peristaltic pump and a flow rate of ∼10 mL/h. An upright Leica SPE TCS Laser Scanning Confocal Microscope (LSCM) was used to image biofilms. GFP was excited at 488 nm, and fluorescence emission was collected in the range of 504–530 nm. Z-stacks of 2D confocal images were rendered in three dimensions using Imaris (Bitplane) (49). Biofilm volumes were quantified using Imaris, by calculating the cumulative volume of each z-stack, and expressed in µm³.

### Antibiotic sensitivity assay

Resistance to the growth inhibitory activity of several antibiotics was assessed by the Kirby-Bauer disc diffusion test. Bacterial cell suspensions in saline were normalized at 0.5 McFarland Standard and swabbed onto MH agar plates supplemented or not with arabinose at the indicated concentration, using disks containing gentamicin (10 µg), kanamycin (30 µg), streptomycin (10 µg), tetracycline (30 µg), ciprofloxacin (5 µg), ceftazidime (30 µg), or imipenem (10 µg) (Becton Dickinson). Growth inhibition halo diameters were measured after 24 h of growth at 37°C.

For time-killing assays experiments in *P. aeruginosa* strains, bacterial cells were diluted at about 5 × 10^6^ CFU/mL in MH in the presence or absence of different antibiotics at 1×, 2×, or 4× MIC. Bacterial cultures were incubated at 37°C and, at different time points, serial dilutions were prepared in saline and plated onto MH agar plates supplemented with 0.5% arabinose for CFU counting. For the same experiments with *E. coli* strains, bacteria were grown overnight in LB and Tc and the morning refreshed 1/100 in LB. The cultures were grown to an OD_600_ of 0.2 (time zero; CFU ± 10^9^) and were each split into two series, one with Gm 5 µg/mL, while the others were left untreated. Cultures were incubated at 37°C and, at different time points, 1:10 serial dilutions were prepared in LB and 5 µL were spotted onto LB plates. The plates were incubated overnight at 37 ˚C before CFU were counted.

### β-Galactosidase activity

β-Galactosidase assays were carried out as previously described (50). Briefly, strains with *hmpA* and *iscR* promoter were refreshed 1:100 in LB supplemented with 100 µM IPTG, after an overnight culture, and β-galactosidase assay was performed when OD_600_ ±2 was reached. Strains with *soxS* promoter were refreshed 1:100 in LB after an overnight culture and then, after 2 h, 100 µM IPTG and 30 µM PMS have been added, and β-galactosidase assays were performed at different time points. Cultures without PMS were used as untreated controls.

### Western blot analysis

Appropriate volumes of bacterial cultures or bacterial suspensions were centrifuged, and pellets were suspended in SDS-PAGE loading buffer (0.25 M Tris-HCl [pH 6.8], 2% SDS, 10% β-mercaptoethanol, 20% glycerol) for SDS-PAGE analysis of whole-cell extracts. Pellets from identical culture volumes were also collected to determine the cellular protein concentration for each sample by using the DC protein assay kit (Bio-Rad) with bovine serum albumin as the standard. Volumes of SDS-PAGE samples corresponding to 20 µg of proteins were loaded onto the gels. Proteins resolved by SDS-PAGE were electrotransferred onto a nitrocellulose filter (Hybond-C extra, Amersham) and probed for IscU, IscS, or LptC using polyclonal rabbit antibodies (51, 52) and a goat anti-rabbit IgG HRP-conjugated secondary antibody (Sigma-Aldrich). Filters were developed with ECL chemiluminescent reagents (Amersham) and visualized on a ChemiDoc XRS + system (Bio-Rad).

### Enzymatic assays

Bacterial cells were resuspended in 30 mM Tris/HCl (pH 8) containing 100 µg/mL lysozyme and lysed by sonication. Cell debris was removed by low-speed centrifugation (5,000 × *g* for 20 min), and the resulting supernatants were used to perform enzymatic assays. SDH and aconitase activities were measured using Succinate Dehydrogenase Assay Kit and the Aconitase Activity Assay Kit (Sigma-Aldrich), respectively. Fumarase A activity was measured as previously described (53). Enzymatic activities were normalized to the protein content of the corresponding cell lysates, determined with the DC protein assay kit (Bio-Rad), and expressed as percentage relative to the wild-type control.

### Statistical analysis

Statistical analysis was performed with the software GraphPad Instat, using one-way analysis of variance (ANOVA) followed by Tukey-Kramer multiple comparison tests.

### Bioinformatic analysis

We downloaded 139 complete reference proteomes of *Pseudomonadales* available on NCBI Assembly in November 2023 and also gathered 62 genomes of *Oceanospirillales* as it corresponds to the sister clade of *Pseudomonadales* (54) (for details, see Supplementary data). The protein data sets of homologs of SUF, ISC, and NIF components built by Garcia et al. (5) were aligned using MAFFT v7.419 (auto option, 55). The alignments were used to build HMM profiles using hmmbuild from HMMER suite v3.2.1 (56). The homologs present in the database were identified using hmmsearch, and sequences presenting an *e*-value <0.01 were selected and aligned. Preliminary phylogenies were inferred using Fasttree v2.1.10 (LG + G4 [57]). The synteny with other components was mapped on trees, and the groups corresponding to the different components were delineated accordingly (supplementary data available on Figshare at 10.6084/m9.figshare.27094924). For the species tree, we used If2, RpoB, and RpoC as phylogenetic markers. We searched the homologs using BLASTP v2.8.1+ (58) starting from *E. coli* sequences. The sequences were aligned and trimmed using BMGE v1.12 (59), and the alignments were concatenated into a supermatrix. A phylogeny was inferred from the supermatrix using IQ-TREE v1.6.12 (60) with LG + R10 model according to the BIC criteria. The robustness of branches was assessed using 1,000 ultrafast bootstrap replicates. The species tree of *Pseudomonadales* was rooted using *Oceanospirillales* mixed with a few genomes annotated as *Pseudomonadales* as an outgroup (supplementary data available on Figshare at 10.6084/m9.figshare.27094924). The distribution of SUF, ISC, and NIF components was then mapped on the tree using iTOL (61).

## SUPPLEMENTAL MATERIAL

Supplemental material mbio.01206-24-s0001.docx Tables S1 and S2 and Figures S1 to S6.

ASM does not own the copyrights to Supplemental Material that may be linked to, or accessed through, an article. The authors have granted ASM a non-exclusive, world-wide license to publish the Supplemental Material files. Please contact the corresponding author directly for reuse.

## References

[1] Kiley PJ, Beinert H. 2003. The role of Fe-S proteins in sensing and regulation in bacteria. Curr Opin Microbiol 6:181–185. doi:10.1016/s1369-5274(03)00039-0 12732309

[2] Mettert EL, Kiley PJ. 2015. Fe-S proteins that regulate gene expression. Biochim Biophys Acta 1853:1284–1293. doi:10.1016/j.bbamcr.2014.11.018 25450978 PMC4390428

[3] Py B, Barras F. 2010. Building Fe-S proteins: bacterial strategies. Nat Rev Microbiol 8:436–446. doi:10.1038/nrmicro2356 20467446

[4] Esquilin-Lebron K, Dubrac S, Barras F, Boyd JM. 2021. Bacterial approaches for assembling iron-sulfur proteins. MBio 12:e0242521. doi:10.1128/mBio.02425-21 34781750 PMC8593673

[5] Garcia PS, D’Angelo F, Ollagnier de Choudens S, Dussouchaud M, Bouveret E, Gribaldo S, Barras F. 2022. An early origin of iron-sulfur cluster biosynthesis machineries before earth oxygenation. Nat Ecol Evol 6:1564–1572. doi:10.1038/s41559-022-01857-1 36109654

[6] Jacobson MR, Cash VL, Weiss MC, Laird NF, Newton WE, Dean DR. 1989. Biochemical and genetic analysis of the nifUSVWZM cluster from Azotobacter vinelandii*.* Mol Gen Genet 219:49–57. doi:10.1007/BF00261156 2615765

[7] Olson JW, Agar JN, Johnson MK, Maier RJ. 2000. Characterization of the NifU and NifS Fe-S cluster formation proteins essential for viability in Helicobacter pylori. Biochemistry 39:16213–16219. doi:10.1021/bi001744s 11123951

[8] Dos Santos PC, Johnson DC, Ragle BE, Unciuleac MC, Dean DR. 2007. Controlled expression of nif and isc iron-sulfur protein maturation components reveals target specificity and limited functional replacement between the two systems. J Bacteriol 189:2854–2862. doi:10.1128/JB.01734-06 17237162 PMC1855825

[9] Outten FW, Djaman O, Storz G. 2004. A suf operon requirement for Fe-S cluster assembly during iron starvation in Escherichia coli. Mol Microbiol 52:861–872. doi:10.1111/j.1365-2958.2004.04025.x 15101990

[10] Ayala-Castro C, Saini A, Outten FW. 2008. Fe-S cluster assembly pathways in bacteria. Microbiol Mol Biol Rev 72:110–125, doi:10.1128/MMBR.00034-07 18322036 PMC2268281

[11] Roche B, Aussel L, Ezraty B, Mandin P, Py B, Barras F. 2013. Iron/sulfur proteins biogenesis in prokaryotes: formation, regulation and diversity. Biochim Biophys Acta 1827:455–469. doi:10.1016/j.bbabio.2012.12.010 23298813

[12] Ezraty B, Vergnes A, Banzhaf M, Duverger Y, Huguenot A, Brochado AR, Su S-Y, Espinosa L, Loiseau L, Py B, Typas A, Barras F. 2013. Fe-S cluster biosynthesis controls uptake of aminoglycosides in a ROS-less death pathway. Science 340:1583–1587. doi:10.1126/science.1238328 23812717

[13] Gerstel A, Zamarreño Beas J, Duverger Y, Bouveret E, Barras F, Py B. 2020. Oxidative stress antagonizes fluoroquinolone drug sensitivity via the SoxR-SUF Fe-S cluster homeostatic axis. PLoS Genet 16:e1009198. doi:10.1371/journal.pgen.1009198 33137124 PMC7671543

[14] Nachin L, El Hassouni M, Loiseau L, Expert D, Barras F. 2001. SoxR-dependent response to oxidative stress and virulence of Erwinia chrysanthemi: the key role of SufC, an orphan ABC ATPase. Mol Microbiol 39:960–972. doi:10.1046/j.1365-2958.2001.02288.x 11251816

[15] Rincon-Enriquez G, Crété P, Barras F, Py B. 2008. Biogenesis of Fe/S proteins and pathogenicity: IscR plays a key role in allowing Erwinia chrysanthemi to adapt to hostile conditions. Mol Microbiol 67:1257–1273. doi:10.1111/j.1365-2958.2008.06118.x 18284573

[16] Vergnes A, Viala JPM, Ouadah-Tsabet R, Pocachard B, Loiseau L, Méresse S, Barras F, Aussel L. 2017. The iron-sulfur cluster sensor IscR is a negative regulator of Spi1 type III secretion system in Salmonella enterica. Cell Microbiol 19. doi:10.1111/cmi.12680 27704705

[17] Huet G, Daffé M, Saves I. 2005. Identification of the Mycobacterium tuberculosis SUF machinery as the exclusive mycobacterial system of [Fe-S] cluster assembly: evidence for its implication in the pathogen’S survival. J Bacteriol 187:6137–6146. doi:10.1128/JB.187.17.6137-6146.2005 16109955 PMC1196142

[18] Roberts CA, Al-Tameemi HM, Mashruwala AA, Rosario-Cruz Z, Chauhan U, Sause WE, Torres VJ, Belden WJ, Boyd JM. 2017. The suf iron-sulfur cluster biosynthetic system is essential in Staphylococcus aureus, and decreased suf function results in global metabolic defects and reduced survival in human neutrophils. Infect Immun 85:e00100-17. doi:10.1128/IAI.00100-17 28320837 PMC5442634

[19] Riboldi GP, Verli H, Frazzon J. 2009. Structural studies of the Enterococcus faecalis SufU [Fe-S] cluster protein. BMC Biochem 10:3. doi:10.1186/1471-2091-10-3 19187533 PMC2644719

[20] Romsang A, Duang-Nkern J, Leesukon P, Saninjuk K, Vattanaviboon P, Mongkolsuk S. 2014. The iron-sulphur cluster biosynthesis regulator IscR contributes to iron homeostasis and resistance to oxidants in Pseudomonas aeruginosa. PLoS ONE 9:e86763. doi:10.1371/journal.pone.0086763 24466226 PMC3899308

[21] Zimbler DL, Park TM, Arivett BA, Penwell WF, Greer SM, Woodruff TM, Tierney DL, Actis LA. 2012. Stress response and virulence functions of the Acinetobacter baumannii NfuA Fe-S scaffold protein. J Bacteriol 194:2884–2893. doi:10.1128/JB.00213-12 22467784 PMC3370640

[22] Stover CK, Pham XQ, Erwin AL, Mizoguchi SD, Warrener P, Hickey MJ, Brinkman FS, Hufnagle WO, Kowalik DJ, Lagrou M, et al.. 2000. Complete genome sequence of Pseudomonas aeruginosa PAO1, an opportunistic pathogen. Nature New Biol 406:959–964. doi:10.1038/35023079 10984043

[23] Saninjuk K, Romsang A, Duang-Nkern J, Wongsaroj L, Leesukon P, Dubbs JM, Vattanaviboon P, Mongkolsuk S. 2023. Monothiol glutaredoxin is essential for oxidative stress protection and virulence in Pseudomonas aeruginosa. Appl Environ Microbiol 89:e0171422. doi:10.1128/aem.01714-22 36533942 PMC9888271

[24] Romsang A, Duang-Nkern J, Saninjuk K, Vattanaviboon P, Mongkolsuk S. 2018. Pseudomonas aeruginosa nfuA: Gene regulation and its physiological roles in sustaining growth under stress and anaerobic conditions and maintaining bacterial virulence. PLoS One 13:e0202151. doi:10.1371/journal.pone.0202151 30092083 PMC6084964

[25] Lopatkin AJ, Stokes JM, Zheng EJ, Yang JH, Takahashi MK, You L, Collins JJ. 2019. Bacterial metabolic state more accurately predicts antibiotic lethality than growth rate. Nat Microbiol 4:2109–2117. doi:10.1038/s41564-019-0536-0 31451773 PMC6879803

[26] Liu Y, Li R, Xiao X, Wang Z. 2019. Bacterial metabolism-inspired molecules to modulate antibiotic efficacy. J Antimicrob Chemother 74:3409–3417. doi:10.1093/jac/dkz230 31211378

[27] Stokes JM, Lopatkin AJ, Lobritz MA, Collins JJ. 2019. Bacterial metabolism and antibiotic efficacy. Cell Metab 30:251–259. doi:10.1016/j.cmet.2019.06.009 31279676 PMC6990394

[28] Sabnis A, Hagart KL, Klöckner A, Becce M, Evans LE, Furniss RCD, Mavridou DA, Murphy R, Stevens MM, Davies JC, Larrouy-Maumus GJ, Clarke TB, Edwards AM. 2021. Colistin kills bacteria by targeting lipopolysaccharide in the cytoplasmic membrane. Elife 10:e65836. doi:10.7554/eLife.65836 33821795 PMC8096433

[29] Visca P, Pisa F, Imperi F. 2019. The antimetabolite 3-bromopyruvate selectively inhibits Staphylococcus aureus. Int J Antimicrob Agents 53:449–455. doi:10.1016/j.ijantimicag.2018.11.008 30472291

[30] Cervoni M, Lo Sciuto A, Bianchini C, Mancone C, Imperi F. 2021. Exogenous and endogenous phosphoethanolamine transferases differently affect colistin resistance and fitness in Pseudomonas aeruginosa. Front Microbiol 12:778968. doi:10.3389/fmicb.2021.778968 34777328 PMC8578941

[31] Imlay JA. 2019. Where in the world do bacteria experience oxidative stress? Environ Microbiol 21:521–530. doi:10.1111/1462-2920.14445 30307099 PMC7301649

[32] da Cruz Nizer WS, Inkovskiy V, Versey Z, Strempel N, Cassol E, Overhage J. 2021. Oxidative stress response in Pseudomonas aeruginosa. Pathogens 10:1187. doi:10.3390/pathogens10091187 34578219 PMC8466533

[33] Schwartz CJ, Djaman O, Imlay JA, Kiley PJ. 2000. The cysteine desulfurase, IscS, has a major role in in vivo Fe-S cluster formation in Escherichia coli. Proc Natl Acad Sci U S A 97:9009–9014. doi:10.1073/pnas.160261497 10908675 PMC16812

[34] Vinella D, Loiseau L, Ollagnier de Choudens S, Fontecave M, Barras F. 2013. In vivo [Fe-S] cluster acquisition by IscR and NsrR, two stress regulators in Escherichia coli. Mol Microbiol 87:493–508. doi:10.1111/mmi.12135 23320508

[35] Vinella D, Brochier-Armanet C, Loiseau L, Talla E, Barras F. 2009. Iron-sulfur (Fe/S) protein biogenesis: phylogenomic and genetic studies of A-type carriers. PLoS Genet 5:e1000497. doi:10.1371/journal.pgen.1000497 19478995 PMC2682760

[36] Liberati NT, Urbach JM, Miyata S, Lee DG, Drenkard E, Wu G, Villanueva J, Wei T, Ausubel FM. 2006. An ordered, nonredundant library of Pseudomonas aeruginosa strain PA14 transposon insertion mutants. Proc Natl Acad Sci U S A 103:2833–2838. doi:10.1073/pnas.0511100103 16477005 PMC1413827

[37] Lee SA, Gallagher LA, Thongdee M, Staudinger BJ, Lippman S, Singh PK, Manoil C. 2015. General and condition-specific essential functions of Pseudomonas aeruginosa*.* Proc Natl Acad Sci U S A 112:5189–5194. doi:10.1073/pnas.1422186112 25848053 PMC4413342

[38] Turner KH, Wessel AK, Palmer GC, Murray JL, Whiteley M. 2015. Essential genome of Pseudomonas aeruginosa in cystic fibrosis sputum. Proc Natl Acad Sci U S A 112:4110–4115. doi:10.1073/pnas.1419677112 25775563 PMC4386324

[39] Poulsen BE, Yang R, Clatworthy AE, White T, Osmulski SJ, Li L, Penaranda C, Lander ES, Shoresh N, Hung DT. 2019. Defining the core essential genome of Pseudomonas aeruginosa. Proc Natl Acad Sci U S A 116:10072–10080. doi:10.1073/pnas.1900570116 31036669 PMC6525520

[40] Lill R, Freibert SA. 2020. Mechanisms of mitochondrial iron-sulfur protein biogenesis. Annu Rev Biochem 89:471–499. doi:10.1146/annurev-biochem-013118-111540 31935115

[41] Lo Sciuto A, Fernández-Piñar R, Bertuccini L, Iosi F, Superti F, Imperi F. 2014. The periplasmic protein TolB as a potential drug target in Pseudomonas aeruginosa. PLoS One 9:e103784. doi:10.1371/journal.pone.0103784 25093328 PMC4122361

[42] Sambrook J, Fritsch EF, Maniatis T. 1989. Molecular cloning: a laboratory manual. 2nd ed. Cold Spring Harbor Laboratory Press, Cold Spring Harbor, New York.

[43] Mdluli KE, Witte PR, Kline T, Barb AW, Erwin AL, Mansfield BE, McClerren AL, Pirrung MC, Tumey LN, Warrener P, Raetz CRH, Stover CK. 2006. Molecular validation of LpxC as an antibacterial drug target in Pseudomonas aeruginosa. Antimicrob Agents Chemother 50:2178–2184. doi:10.1128/AAC.00140-06 16723580 PMC1479155

[44] Milton DL, O’Toole R, Horstedt P, Wolf-Watz H. 1996. Flagellin A is essential for the virulence of Vibrio anguillarum. J Bacteriol 178:1310–1319. doi:10.1128/jb.178.5.1310-1319.1996 8631707 PMC177804

[45] Hoang TT, Kutchma AJ, Becher A, Schweizer HP. 2000. Integration-proficient plasmids for Pseudomonas aeruginosa: site-specific integration and use for engineering of reporter and expression strains. Plasmid 43:59–72. doi:10.1006/plas.1999.1441 10610820

[46] Lo Sciuto A, Spinnato MC, Pasqua M, Imperi F. 2022. Generation of stable and unmarked conditional mutants in Pseudomonas aeruginosa. Methods Mol Biol 2548:21–35. doi:10.1007/978-1-0716-2581-1_2 36151489

[47] Heeb S, Blumer C, Haas D. 2002. Regulatory RNA as mediator in GacA/RsmA-dependent global control of exoproduct formation in Pseudomonas fluorescens CHA0. J Bacteriol 184:1046–1056. doi:10.1128/jb.184.4.1046-1056.2002 11807065 PMC134805

[48] Pasqua M, Visaggio D, Lo Sciuto A, Genah S, Banin E, Visca P, Imperi F. 2017. Ferric uptake regulator fur is conditionally essential in Pseudomonas aeruginosa*.* J Bacteriol 199:e00472-17. doi:10.1128/JB.00472-17 28847923 PMC5648857

[49] Cohen D, Mechold U, Nevenzal H, Yarmiyhu Y, Randall TE, Bay DC, Rich JD, Parsek MR, Kaever V, Harrison JJ, Banin E. 2015. Oligoribonuclease is a central feature of cyclic diguanylate signaling in Pseudomonas aeruginosa. Proc Natl Acad Sci U S A 112:11359–11364. doi:10.1073/pnas.1421450112 26305928 PMC4568660

[50] Miller JH. 1992. A short course in bacterial genetics. In A laboratory manual and handbook for Escherichia coli and related bacteria. Cold Spring Harbor Laboratory Press.

[51] Kato S, Mihara H, Kurihara T, Takahashi Y, Tokumoto U, Yoshimura T, Esaki N. 2002. Cys-328 of IscS and Cys-63 of IscU are the sites of disulfide bridge formation in a covalently bound IscS/IscU complex: implications for the mechanism of iron-sulfur cluster assembly. Proc Natl Acad Sci U S A 99:5948–5952. doi:10.1073/pnas.082123599 11972033 PMC122882

[52] Lo Sciuto A, Martorana AM, Fernández-Piñar R, Mancone C, Polissi A, Imperi F. 2018. Pseudomonas aeruginosa LptE is crucial for LptD assembly, cell envelope integrity, antibiotic resistance and virulence. Virulence 9:1718–1733. doi:10.1080/21505594.2018.1537730 30354941 PMC7204523

[53] Gort AS, Imlay JA. 1998. Balance between endogenous superoxide stress and antioxidant defenses. J Bacteriol 180:1402–1410. doi:10.1128/JB.180.6.1402-1410.1998 9515906 PMC107037

[54] Handley KM, Lloyd JR. 2013. Biogeochemical implications of the ubiquitous colonization of marine habitats and redox gradients by Marinobacter species. Front Microbiol 4:136. doi:10.3389/fmicb.2013.00136 23734151 PMC3660661

[55] Katoh K, Standley DM. 2013. MAFFT multiple sequence alignment software version 7: improvements in performance and usability. Mol Biol Evol 30:772–780. doi:10.1093/molbev/mst010 23329690 PMC3603318

[56] Eddy SR. 2011. Accelerated profile HMM searches. PLoS Comput Biol 7:e1002195. doi:10.1371/journal.pcbi.1002195 22039361 PMC3197634

[57] Price MN, Dehal PS, Arkin AP. 2010. FastTree 2--approximately maximum-likelihood trees for large alignments. PLoS One 5:e9490. doi:10.1371/journal.pone.0009490 20224823 PMC2835736

[58] Altschul SF, Madden TL, Schäffer AA, Zhang J, Zhang Z, Miller W, Lipman DJ. 1997. Gapped BLAST and PSI-BLAST: a new generation of protein database search programs. Nucleic Acids Res 25:3389–3402. doi:10.1093/nar/25.17.3389 9254694 PMC146917

[59] Criscuolo A, Gribaldo S. 2010. BMGE (Block Mapping and Gathering with Entropy): a new software for selection of phylogenetic informative regions from multiple sequence alignments. BMC Evol Biol 10:210. doi:10.1186/1471-2148-10-210 20626897 PMC3017758

[60] Nguyen L-T, Schmidt HA, von Haeseler A, Minh BQ. 2015. IQ-TREE: a fast and effective stochastic algorithm for estimating maximum-likelihood phylogenies. Mol Biol Evol 32:268–274. doi:10.1093/molbev/msu300 25371430 PMC4271533

[61] Letunic I, Bork P. 2019. Interactive tree of life (iTOL) v4: recent updates and new developments. Nucleic Acids Res 47:W256–W259. doi:10.1093/nar/gkz239 30931475 PMC6602468

