# Supplementary material for: A molecular comparison of [Fe-S] cluster-based homeostasis in Escherichia coli and Pseudomonas aeruginosa
Source: mBio. 2024 Oct 3;15(11):e01206-24. doi: 10.1128/mbio.01206-24 (PMC11559095; doi:10.1128/mbio.01206-24)
Supplement: Supplemental material — Tables S1 and S2 and Figures S1 to S6. [file mbio.01206-24-s0001.docx]

**A molecular comparison of [Fe-S] cluster-based homeostasis in *Escherichia coli* and *Pseudomonas aeruginosa***

Alessandra Lo Sciuto, Francesca D’Angelo, Maria Concetta Spinnato, Pierre Simon Garcia, Shirley Genah, Emmanuel Séchet, Ehud Banin, Frédéric Barras, and Francesco Imperi

**SUPPLEMENTARY MATERIAL**

Table S1. Bacterial strains and plasmids used in this study.

Table S2. Primers used in this study.

Figure S1. Survival curves of IscU-replete and -depleted *P. aeruginosa* exposed to antibiotics at 1× or 2×MIC.

Figure S2. Antibiotic sensitivity of ISC-deficient *P. aeruginosa* expressing *E. coli* SUF.

Figure S3. Deletion of *iscU* is viable in *P. aeruginosa* expressing *E. coli* SUF.

Figure S4. Western blot analysis to evaluate IPTG-dependent expression from the plasmid pME_iscEC_.

Figure S5. Colony growth of ISC-deficient *P. aeruginosa* expressing *E. coli* SUF in the presence of H_2_O_2_ at 0.5× or 1×MIC.

Figure S6. Planktonic growth of *E. coli* MG1655 and *P. aeruginosa* PAO1 in the presence of different H_2_O_2_ concentrations.

**Table S1.** Bacterial strains and plasmids used in this study.

| Strain or plasmid | Genotype and/or relevant characteristics | Reference or source |  |  |
| --- | --- | --- | --- | --- |
| ***P. aeruginosa*** |  |  | |  |
| PAO1 (ATCC15692) | Prototroph WT strain | American type culture collection | | |
| PAO1 *araC*P_araBAD_*iscU* | PAO1 with an arabinose-inducible additional copy of the *iscU* coding sequence (*iscU*_PA_) inserted into the *attB* neutral site | This work | | |
| PAO1 ΔiscU *araC*P_araBAD_*iscU* | PAO1 *araC*P_araBAD_*iscU* with an in-frame deletion of the endogenous copy of *iscU*_PA_ (ΔiscU P_ara_*iscU*) | This work | | |
| PAO1 ΔiscU pMEisc_EC_ | PAO1 carrying the plasmid pMEisc_EC_ for IPTG-induced expression of the *E. coli iscSUAhscBAfdx* operon and with an in-frame deletion of the endogenous copy of *iscU*_PA_ | This work | | |
| PAO1 ΔiscU pMEsuf_EC_ | PAO1 carrying the plasmid pMEsuf_EC_ for IPTG-induced expression of the *E. coli sufABCDSE* operon and with an in-frame deletion of the endogenous copy of *iscU*_PA_ | This work | | |
| ***E. coli*** |  |  | | |
| DH5αF’ | *recA1 endA1 hsdR17 supE44 thi-1 gyrA96 relA1* Δ(*lacZYA-argF*)U169[φ*80 dlacZ*Δ*M15*], Nal^R^ | Liss 1987 | | |
| S17.1λ*pir* | *thi pro hsdRhsdM^+^recA RP4-2-Tc::Mu-Km::Tn7 λpir,* Sm^R^ | Simon et al. 1983 | | |
| MG1655 | *E. coli* WT strain | Lab collection | | |
| MG1655 P*iscR::lacZ* | *E. coli* WT strain containing the transcriptional fusion between *iscR* promoter and *lacZ* gene | Vinella et al. 2013 | | |
| MG1655 P*soxS::lacZ* | *E. coli* WT strain containing the transcriptional fusion between *soxS* promoter and *lacZ* gene | Ezraty et al. 2014 | | |
| MG1655 P*hmpA::lacZ* | *E. coli* WT strain containing the transcriptional fusion between *hmpA* promoter and *lacZ* gene | Vinella et al. 2013 | | |
| MG1655 ΔiscU P*iscR::lacZ* | *E. coli* strain with an in-frame deletion of *iscU.* The strain contains the transcriptional fusion between *iscR* promoter and *lacZ* gene | Roche et al. 2015 | | |
| MG1655 ΔiscU P*hmpA::lacZ* | *E. coli* strain with an in-frame deletion of *iscU.* The strain contains the transcriptional fusion between *hmpA* promoter and *lacZ* gene | Vinella et al. 2013 | | |
| MG1655 ΔiscUA | *E. coli* strain with an in-frame deletion of *iscUA* | Vinella et al. 2009 | | |
| MG1655 Δisc | *E. coli* strain with a deletion of the genes *iscSUAhscBAfdx* | Kindly provided by Beatrice Py | | |
| MG1655 Δsuf | *E. coli* strain with a deletion of the entire *sufABCDSE* operon | Nachin et al. 2003 | | |
| MG1655 Δsuf PsoxS::lacZ | *E. coli* strain with a deletion of the entire *sufABCDSE* operon. The strain contains the transcriptional fusion between *soxS* promoter and *lacZ* gene | Gerstel et al. 2020 | | |
| MG1655 ΔiscUA Δsuf MVA^+^ | *E. coli* strain with an in-frame deletion of *iscUA* and of the entire *sufABCDSE* operon, carrying the MVA cassette (Kn^R^) in the chromosome | Vinella et al. 2009 | | |
| **Plasmid** |  |  | | |
| pBluescript II KS+ | Cloning vector; ColE1 replicon; Ap^R^ | Stratagene | | |
| pDM4 | Suicide vector; *sacB*, *oriR6K*; Cm^R^ | Milton et al. 1996 | | |
| pDM4Δ*iscU* | pDM4 derivative for *iscU*_PA_ in-frame deletion | This work | | |
| mini-CTX1 | Self-proficient integration vector with *tet,* Ω-*FRT*-*attP*-MCS, *ori, int, oriT*; Tc^R^ | Hoang et al. 2000 | | |
| mini-CTX1*-araC*P_araBAD_*tolB* | mini-CTX1 derivative carrying *araC*P_araBAD_*tolB*, Tc^R^ | Lo Sciuto et al. 2014 | | |
| mini-CTX1-*araC*P_araBAD_*iscU* | mini-CTX1-*araC*P_araBAD_*tolB* derivative in which *tolB* has been replaced with the *iscU*_PA_ coding sequence | This work | | |
| pME6032 | IPTG inducible expression vector, *lacI^q^-*P_tac_, Tc^R^ | Heeb et al. 2002 | | |
| pME*iscU*_EC_ | pME6032-derivative carrying the coding sequence of *E. coli iscU* (*iscU*_EC_) downstream of the IPTG-inducible P_tac_ promoter | This work | | |
| pME*isc*_EC_ | pME6032-derivative carrying the *E. coli iscSUAhscBAfdx* operon without the endogenous promoter (*isc*_EC_) downstream of the IPTG-inducible P_tac_ promoter | This work | | |
| pME*suf*_EC_ | pME6032-derivative carrying the *E. coli sufABCDSE* operon without the endogenous promoter (*suf*_EC_) downstream of the IPTG-inducible P_tac_ promoter | This work | | |
| pME*sufBCD*_EC_ | pME6032-derivative carrying the coding sequences of the *E. coli sufBCD* genes (*sufBCD*_EC_) downstream of the IPTG-inducible P_tac_ promoter | This work | | |
| pME*isc*_PA_ | pME6032-derivative carrying the *P. aeruginosa iscSUAhscBAfdx2* operon without the endogenous promoter (*isc*_PA_) downstream of the IPTG-inducible P_tac_ promoter | This work | | |
| pMRP9-1 | pUCP18-derivative which constitutively expresses the GFP protein, Cb^R^ | Davies et al. 1998 | | |

**Supplementary references**

1. Liss L. New M13 host: DH5 F' competent cells. Focus. 1987;9:13.
2. Simon R, Priefer U, Pühler A. A Broad Host Range Mobilization System for In Vivo Genetic Engineering: Transposon Mutagenesis in Gram Negative Bacteria. Nat Biotechnol. 1983;1:784-91 doi: 10.1038/nbt1183-784.
3. Vinella D, Loiseau L, Ollagnier de Choudens S, Fontecave M, Barras F. In vivo [Fe-S] cluster acquisition by IscR and NsrR, two stress regulators in Escherichia coli. Mol Microbiol. 2013;87: 493–508. doi:10.1111/mmi.12135.
4. Ezraty B, Henry C, Hérisse M, Denamur E, Barras F. Commercial Lysogeny Broth culture media and oxidative stress: a cautious tale. Free Radic Biol Med. 2014;74: 245–251. doi:10.1016/j.freeradbiomed.2014.07.010.
5. Roche B, Agrebi R, Huguenot A, Ollagnierde Choudens S, Barras F, Py B. TurningEscherichia coli into a Frataxin-Dependent Organism. PLoS Genet 2015;11(5): e1005134. doi:10.1371/journal. pgen.1005134.
6. Vinella D, Brochier-Armanet C, Loiseau L, Talla E, Barras F. Iron-sulfur (Fe/S) protein biogenesis: phylogenomic and genetic studies of A-type carriers. PLoS Genet. 2009;5: e1000497. doi:10.1371/journal.pgen.1000497
7. Nachin L, Loiseau L, Expert D, Barras F. SufC: an unorthodox cytoplasmic ABC/ATPase required for [Fe-S] biogenesis under oxidative stress. EMBO J. 2003;22: 427–437. doi:10.1093/emboj/cdg061.
8. Gerstel A, Zamarreño Beas J, Duverger Y, Bouveret E, Barras F, Py B. Oxidative stress antagonizes fluoroquinolone drug sensitivity via the SoxR-SUF Fe-S cluster homeostatic axis. PLoS Genet 2020;16(11): e1009198. https://doi.org/10.1371/journal.pgen.1009198.
9. Milton DL, O'Toole R, Horstedt P, Wolf-Watz H. Flagellin A is essential for the virulence of *Vibrio anguillarum*. J Bacteriol. 1996;178:1310-9. doi: 10.1128/jb.178.5.1310-1319.1996.
10. Hoang TT, Kutchma AJ, Becher A, Schweizer HP. Integration-proficient plasmids for *Pseudomonas aeruginosa*: site-specific integration and use for engineering of reporter and expression strains. Plasmid. 2000;43:59-72. doi: 10.1006/plas.1999.1441.
11. Lo Sciuto A, Fernández-Piñar R, Bertuccini L, Iosi F, Superti F, Imperi F. The Periplasmic Protein TolB as a Potential Drug Target in *Pseudomonas aeruginosa*. PLoS ONE 2014;9(8): e103784. https://doi.org/10.1371/journal.pone.0103784.
12. Heeb S, Blumer C, Haas D. Regulatory RNA as mediator in GacA/RsmA-dependent global control of exoproduct formation in *Pseudomonas fluorescens* CHA0. J Bacteriol. 2002;184:1046-56. doi: 10.1128/jb.184.4.1046-1056.2002
13. Davies DG, Parsek MR, Pearson JP, Iglewski BH, Costerton JW, Greenberg EP. The involvement of cell-to-cell signals in the development of a bacterial biofilm. Science. 1998;280:295-8. doi: 10.1126/science.280.5361.295.

**Table S2.** Primers used in this study.^a^

| Primer name | **Sequence (5’→3’)^b^** | **Restriction site** | **Application** |
| --- | --- | --- | --- |
| *iscU*_PA__FW | cgcaagcttATGGCATATAGCGAAAAGGTC | HindIII | Generation of mini-CTX1-*araC*P_araBAD_*iscU* |
| *iscU*_PA__RV | cggaatTCCTTACTCGTCACGAGCG | EcoRI |  |
| *iscU*_PA_ del_UP_FW | ccgctCGAGGCGTTCCGCATCGC | XhoI | Generation of pDM4Δ*iscU*_PA_ |
| *iscU*_PA_ del_UP_RV | cgggatcCTTGCCGACGTTGCGCGG | BamHI |  |
| *iscU*_PA_ del_DOWN_FW | cgggatcCCATCAAGGCCGCCGTTC | BamHI |  |
| *iscU*_PA_ del_DOWN_RV | gctctAGACGCTGCTCGCGCTCG | XbaI |  |
| *iscU*_EC__FW | cggaattcCGGTATCGGAATCAGGAG | EcoRI | Generation of pME*iscU*_EC_ |
| *iscU*_EC__RV | ccgctcgaGTGTAATCGACATAACCAAACC | XhoI |  |
| *iscSUAhscBAfdx2*_PA__FW | gaagatctCCAGCCTGTAGGAGTTACC | BglII | Generation of pME*isc*_PA_ |
| *iscSUAhscBAfdx2*_PA__RV | ggaagatCTCTACTACCGCCGGGC | BglII |  |
| *iscSUAhscBAfdxB*_EC__FW | cggaattCGGTAGCCTGATTCCTTG | EcoRI | Generation of pME*isc*_EC_ |
| *iscSUAhscBAfdxB*_EC__RV | ccgctcgaGGTCCACTTAAGTCCCATAC | XhoI |  |
| *sufABCDSE*_FW | cggaattCTGAAAGCGATGAAGTGAGG | EcoRI | Generation of pME*suf*_EC_ |
| *sufABCDSE*_RV | ccgctcGAGCCAACCGGATGAAAGC | XhoI |  |
| *sufBCD*_FW | cggaattCTGTGGCGAAAGCTTTG | EcoRI | Generation of pME*sufBCD*_EC_ |
| *sufBCD*_RV | ccgctcgagCTCACGCGAAAGCACC | XhoI |  |
| M13FW | GTTTTCCCAGTCACGAC |  | Sequencing of pBS constructs |
| M13RV | CAGGAAACAGCTATGAC |  |  |
| P_araBAD__FW | CATAAGATTAGCGGATCCTAC |  | Sequencing of mini-CTX1*-araC*P_araBAD_ constructs |
| pME6032_FW | GCTCTCGGGTAACATCAAG |  | Sequencing of pME6032 constructs |
| pME6032_RV | CGGTTCTGGCAAATATTCTG |  |  |
| *sufABCDSE* walking_1 | TAAGTAAAGAGGTGGAGGCG |  | Sequencing of the *suf*_EC_ and *sufBCD*_EC_ inserts |
| *sufABCDSE* walking_2 | GCAATTTCACTCAGTGCGAC |  |  |
| *sufABCDSE* walking_3 | CTTGATGAGTCGGACTCCG |  |  |
| *sufABCDSE* walking_4 | TTCACTATCAACGTCGCAGC |  |  |
| *sufABCDSE* walking_5 | AAGCGCCCAGGCGACCG |  |  |
| *iscSUAhscBAfdxB*_EC_ walking_1 | GTTCACCAGATCGTCGG |  | Sequencing of the *isc*_EC_ insert |
| *iscSUAhscBAfdxB*_EC_ walking_2 | CATACCGATAACCCCACC |  |  |
| *iscSUAhscBAfdxB*_EC_ walking_3 | GCTCGATACCGCCAAC |  |  |
| *iscSUAhscBAfdxB*_EC_ walking_4 | CGGTTCTACTCGCGTG |  |  |
| *iscSUAhscBAfdx2*_PA_ walking_1 | CACCCTAGCCACCCAC |  | Sequencing of the *isc*_PA_ insert |
| *iscSUAhscBAfdx2*_PA_ walking_2 | CGAGGCCTACCAGACC |  |  |
| *iscSUAhscBAfdx2*_PA_ walking_3 | GCTACCAAGGATGCCG |  |  |

^a^ Preparative PCRs for cloning were performed using the genomic DNA of *P. aeruginosa* PAO1 or that of *E. coli* MG1655 as the template.

^b^ The restriction site used for cloning is underlined in the primer sequence.

**Figure S1**. Survival curves of IscU-replete and -depleted cells, obtained as described in Figure 1C, exposed to the indicated antibiotics at 1×MIC (A) or 2×MIC (B). Data are the mean (±SD) of at least three independent assays.

**Figure S2.** Survival curves of the *P. aeruginosa* conditional mutant ΔiscU P_ara_*iscU* carrying pME*suf_EC_,* and of PAO1 pME6032 and PAO1 pME*suf_EC_* as controls*,* cultured in the presence of 0.5 mM IPTG and exposed to the indicated antibiotics at 4×MIC. Data are the mean (±SD) of three independent assays.

**Figure S3**. The *E. coli* SUF system functionally replaces *P. aeruginosa* ISC. (A) Colony and (B) planktonic growth of the *P. aeruginosa* PAO1 *iscU* deletion mutant (Δ*iscU*) carrying the plasmid pME6032 with the entire *suf* operon from *E. coli* (*suf*_EC_) or the entire *E. coli isc* operon (*isc*_EC_) in the absence or presence of 0.5 mM IPTG. PAO1 with the empty plasmid pME6032 was used as the positive control. Images and graphs are representative of three independent assays.


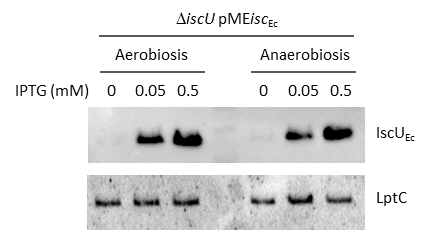


**Figure S4**. Western blot analysis for IscU and the loading control LptC in the *P. aeruginosa* deletion mutant ΔiscU carrying the plasmid pME*isc*_EC_ cultured on MH agar plates, supplemented or not with IPTG at 0.05 and 0.5 mM, under aerobic or anaerobic conditions. Whole-cell extracts were prepared from cells recovered from plates and resuspended in saline. Images are representative of two independent assays.


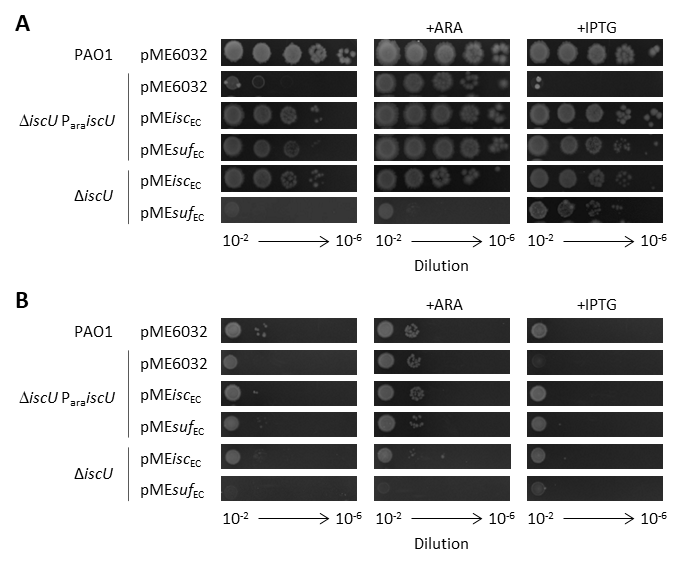


**Figure S5**. Colony growth of the *P. aeruginosa* conditional mutant ΔiscU P_ara_*iscU* or the Δ*iscU* deletion mutant carrying the plasmid pME6032 with the entire *isc* operon *(isc*_EC_) or the entire *suf* operon (*suf*_EC_) of *E. coli* under an IPTG-inducible promoter in the presence of H_2_O_2_ at **(A)** 0.5×MIC or **(B)** 1×MIC for the wild type (corresponding to 0.5 mM and 1 mM, respectively). PAO1 and ΔiscU P_ara_*iscU* with the empty plasmid pME6032 were used as positive and negative controls, respectively. When indicated, arabinose (ARA) and IPTG were added at 0.5% and 0.5 mM, respectively. Images are representative of three independent assays.

**
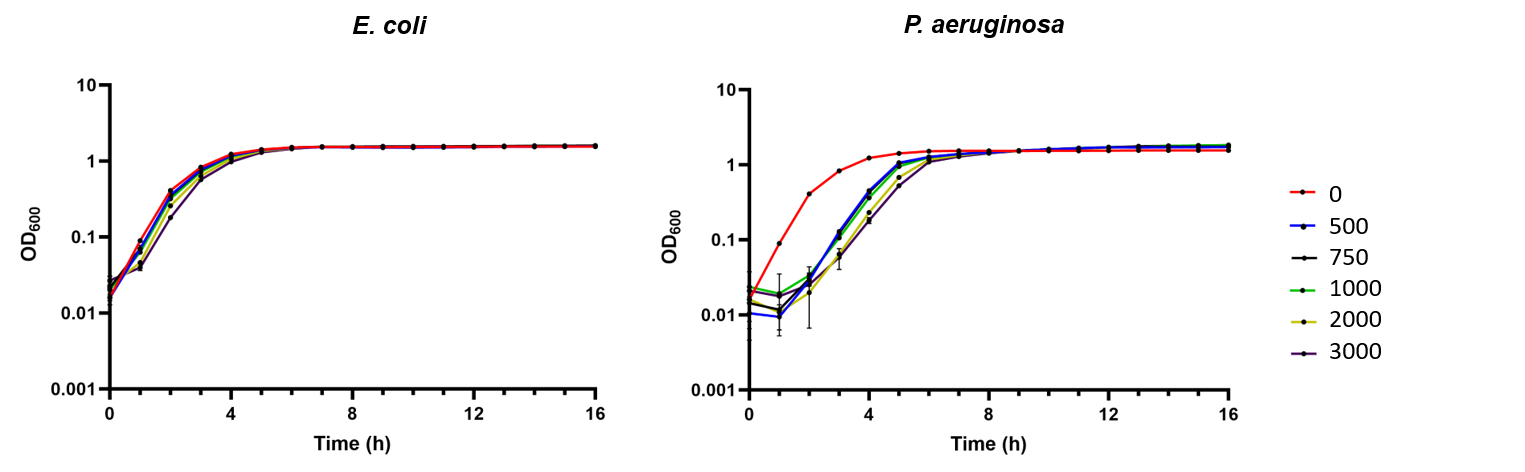
**

**Figure S6**. Planktonic growth of *E. coli* MG1655 and *P. aeruginosa* PAO1 in LB in the absence or presence of the H_2_O_2_ concentrations (expressed in µM) indicated in the graph. Data are the mean (±SD) of three independent assays.
